# MiR-155 Enhances Insulin Sensitivity by Coordinated Regulation of Multiple Genes in Mice

**DOI:** 10.1371/journal.pgen.1006308

**Published:** 2016-10-06

**Authors:** Xiaolin Lin, Yujuan Qin, Junshuang Jia, Taoyan Lin, Xia Lin, Li Chen, Hui Zeng, Yanjiang Han, Lihong Wu, Shun Huang, Meng Wang, Shenhao Huang, Raoying Xie, Liqi Liang, Yu Liu, Ruiyu Liu, Tingting Zhang, Jing Li, Shengchun Wang, Penghui Sun, Wenhua Huang, Kaitai Yao, Kang Xu, Tao Du, Dong Xiao

**Affiliations:** 1 Guangdong Provincial Key Laboratory of Cancer Immunotherapy Research and Guangzhou Key Laboratory of Tumor Immunology Research, Cancer Research Institute, Southern Medical University, Guangzhou, China; 2 Department of Endocrinology, The Second Affiliated Hospital, Guangzhou Medical University, Guangzhou, China; 3 Department of Medical Imaging Center, Nanfang Hospital, Southern Medical University, Guangzhou, China; 4 NanFang PET Center, Nanfang Hospital, Southern Medical University, Guangzhou, China; 5 Key Laboratory of Oral Medicine, Guangzhou Institute of Oral Disease, Stomatology Hospital of Guangzhou Medical University, Guangzhou, China; 6 Department of Anatomy, Guangdong Provincial Key Laboratory of Construction and Detection in Tissue Engineering, School of Basic Medical Science, Southern Medical University, Guangzhou, China; 7 Department of General Surgery, Sun Yat-sen Memorial Hospital, Sun Yat-sen University, Guangzhou, China; 8 Guangdong Provincial Key Laboratory of Malignant Tumor Epigenetics and Gene Regulation, Sun Yat-Sen Memorial Hospital, Sun Yat-Sen University, Guangzhou, China; 9 Institute of Comparative Medicine & Laboratory Animal Center, Southern Medical University, Guangzhou, China; University of California San Francisco, UNITED STATES

## Abstract

miR-155 plays critical roles in numerous physiological and pathological processes, however, its function in the regulation of blood glucose homeostasis and insulin sensitivity and underlying mechanisms remain unknown. Here, we reveal that miR-155 levels are downregulated in serum from type 2 diabetes (T2D) patients, suggesting that miR-155 might be involved in blood glucose control and diabetes. Gain-of-function and loss-of-function studies in mice demonstrate that miR-155 has no effects on the pancreatic β-cell proliferation and function. Global transgenic overexpression of miR-155 in mice leads to hypoglycaemia, improved glucose tolerance and insulin sensitivity. Conversely, miR-155 deficiency in mice causes hyperglycemia, impaired glucose tolerance and insulin resistance. In addition, consistent with a positive regulatory role of miR-155 in glucose metabolism, miR-155 positively modulates glucose uptake in all cell types examined, while mice overexpressing miR-155 transgene show enhanced glycolysis, and insulin-stimulated AKT and IRS-1 phosphorylation in liver, adipose tissue or skeletal muscle. Furthermore, we reveal these aforementioned phenomena occur, at least partially, through miR-155-mediated repression of important negative regulators (i.e. C/EBPβ, HDAC4 and SOCS1) of insulin signaling. Taken together, these findings demonstrate, for the first time, that miR-155 is a positive regulator of insulin sensitivity with potential applications for diabetes treatment.

## Author Summary

In the present study, we provide evidence for the first time showing that miR-155 is a positive regulator of insulin sensitivity in mice. Here, we determine that miR-155 levels are downregulated in serum from type 2 diabetes (T2D) patients, and shows a negative correlation with HOMA-IR, suggesting that miR-155 might be involved in glucose homeostasis and insulin action. Global transgenic overexpression of miR-155 in mice leads to hypoglycaemia, improved glucose tolerance and insulin sensitivity. Conversely, miR-155 deficiency in mice causes hyperglycemia, impaired glucose tolerance and insulin resistance. In addition, consistent with a positive regulatory role of miR-155 in glucose metabolism, miR-155 positively modulates glucose uptake in all cell types examined, while mice overexpressing miR-155 transgene show enhanced glycolysis, and insulin-stimulated AKT and IRS-1 phosphorylation in liver, adipose tissue or skeletal muscle. More importantly, we reveal that these aforementioned phenomena occur, at least in part, through the miR-155-mediated coordinated repression of multiple negative regulators (i.e. C/EBPβ, HDAC4 and SOCS1) of insulin signaling.

## Introduction

Diabetes is recognized as one of the most important health threats of our time[1–4]. However, the major mechanisms underlying the pathogenesis of diabetes remain unclear. microRNAs (miRNAs) are involved in glucose homeostasis, insulin sensitivity and pancreatic β-cell function, and the pathogenesis of diabetes[1–4]. miR-375 and miR-34a are associated with pancreatic development, and miR-375 and miR-9 are implicated in insulin secretion[1, 3]. miRNAs, including miR-103/miR-107, miR-143 and miR-802, have been confirmed to be negative regulators of insulin sensitivity in intact animals[1, 3]. However, much work remains to be done to discover miRNAs that play positive roles in regulating insulin sensitivity and glucose metabolism, which provides us a better understanding of the functions of these miRNAs in modulating blood glucose homeostasis and greatly helps us find more potential treatment targets.

As a multifunctional miRNA, miR-155 plays crucial roles in various physiological and pathological processes, such as haematopoietic lineage differentiation, cardiovascular diseases and cancer[5–7]. Our report showed that Rm155LG/Alb-Cre transgenic mice with liver-specific miR-155 overexpression exhibited the reduced levels of hepatic and serum lipid compositions[8]. In pilot experiment, we found that miR-155 levels in serum of type 2 diabetes (T2D) patients were lower than in healthy subjects, suggesting that miR-155 might be involved in blood glucose control and diabetes, which remains to be fully explored.

In the present study, we investigate the roles of miR-155 in blood glucose homeostasis, as well as the underlying mechanisms. Our findings show, for the first time, that miR-155 enhances insulin sensitivity through coordinated regulation of multiple genes in mice, including important negative regulators (i.e. C/EBPβ, HDAC4 and SOCS1) of insulin signaling.

## Results

### Dysregulated miR-155 levels in serum from T2D patients

**Fig 1:**
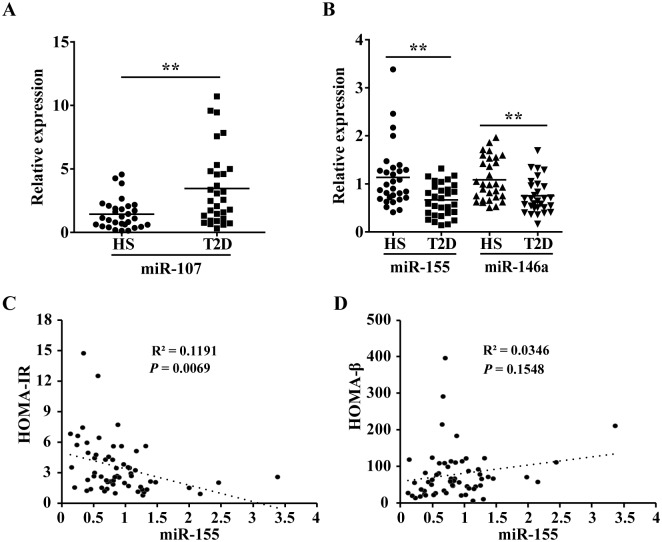
Dysregulated miR-155 levels in serum from T2D patients. **(A-B)** Basal levels of miR-107(A), miR-155 (B) and miR-146a (B) in healthy subjects (HS) (n = 30) and T2D patients (n = 30) detected by qRT-PCR. Values are statistically significant at **P<0.01. **(C-D)** miR155 levels and homeostasis model indicators (HOMA-IR and HOMA-β). HOMA-IR: homeostasis model assessment of insulin resistance; HOMA-β: homeostasis model assessment of β-cell function.

We evaluated the levels of miRNAs in serum of T2D patients. miR-146a levels were decreased in serum[9] and peripheral blood mononuclear cells (PBMCs)[10, 11] from T2D patients, while hepatic overexpression of miR-107 induced hyperglycaemia and insulin resistance in mice[12]. Thus, miR-146a and miR-107 are chosen as references to evaluate the serum miR-155 levels in T2D patients. qRT-PCR analysis revealed a higher levels of miR-107 (Fig 1A) and a lower levels of miR-155 and miR-146a (Fig 1B) in serum from patients with type 2 diabetes mellitus (T2DM). Moreover, miR-155 levels showed negative correlation with HOMA-IR (R2 = 0.1191, *P* = 0.0069, Fig 1C) and no statistically significant correlation with HOMA-β (R2 = 0.0346, *P* = 0.1548, Fig 1D). Together, our observations strongly support that miR-155 might be involved in glucose homeostasis and insulin action.

### Global overexpression of miR-155 transgene in RL-m155 transgenic mice

As described in the Materials and Methods section, to examine the roles of miR-155 by gain of function, Rm155LG transgenic mice (i.e., Rm155LG mice) for the conditional overexpression of mouse miR-155 transgene mediated by Cre/lox P system were generated by us[8].

**Fig 2:**
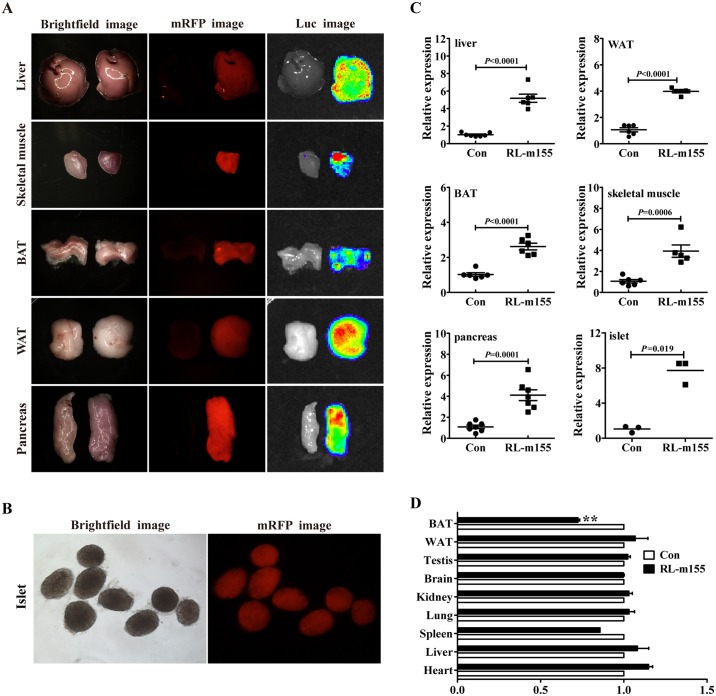
Global overexpression of miR-155 transgene in RL-m155 mice. **(A)** mRFP and luciferase (Luc) expression in multiple organs and tissues of RL-m155 mice. The left and right organ samples in each figure were obtained from control and RL-m155 mice, respectively. In this study, the non-transgenic littermates (i.e., wild-type littermates) were used as controls of RL-m155 transgenic mice. WAT: white adipose tissue; BAT: brown adipose tissue. Other details were described previously[8]. **(B)** mRFP expression in the isolated islets of RL-m155 mice. **(C)** qRT-PCR analysis of miR-155 transgene expression in organs and tissues of RL-m155 mice. **(D)** Relative organ weight of RL-m155 mice vs. control mice.

To achieve global overexpression of miR-155, we crossed Rm155LG mice with EIIa-Cre mice. Procedure for producing RL-m155 transgenic mice (i.e., RL-m155 mice) which can globally overexpress miR-155 transgene and express mRFP and luciferase (Luc) reporter transgenes in multiple organs and tissues is detailedly illustrated in S1A, S1D and S1E Fig exhibited whole-body fluorescence (b) and bioluminescence (c) imaging for newborn (S1D Fig) and adult RL-m155 mice (S1E Fig), respectively. We next determined the expression pattern of the mRFP and Luc transgenes in multiple organs and tissues taken from RL-m155 mice. Both red fluorescence and Luc signals were detected in tissue/organ samples including liver, skeletal muscle (SM), brown adipose tissue (BAT), white adipose tissue (WAT) and pancreas isolated from RL-m155 mice, but not in control littermates (Fig 2A and S2A Fig). Fig 2B demonstrated mRFP expression in the isolated islets of RL-m155 mice. qRT-PCR exhibited significant increases in miR-155 expression levels in liver, WAT, BAT, SM, pancreas and isolated islets of RL-m155 mice (Fig 2C and S2B Fig). Additionally, global overexpression of miR-155 transgene did not alter the final body weight of RL-m155 mice (S2C–S2F Fig). RL-m155 mice displayed significantly reduced weight (Fig 2D) of BAT, compared with their control mice.

### RL-m155 mice and miR-155 knockout (KO) mice exhibit the unaltered β-cell proliferation and hormone profiles in pancreas

**Fig 3:**
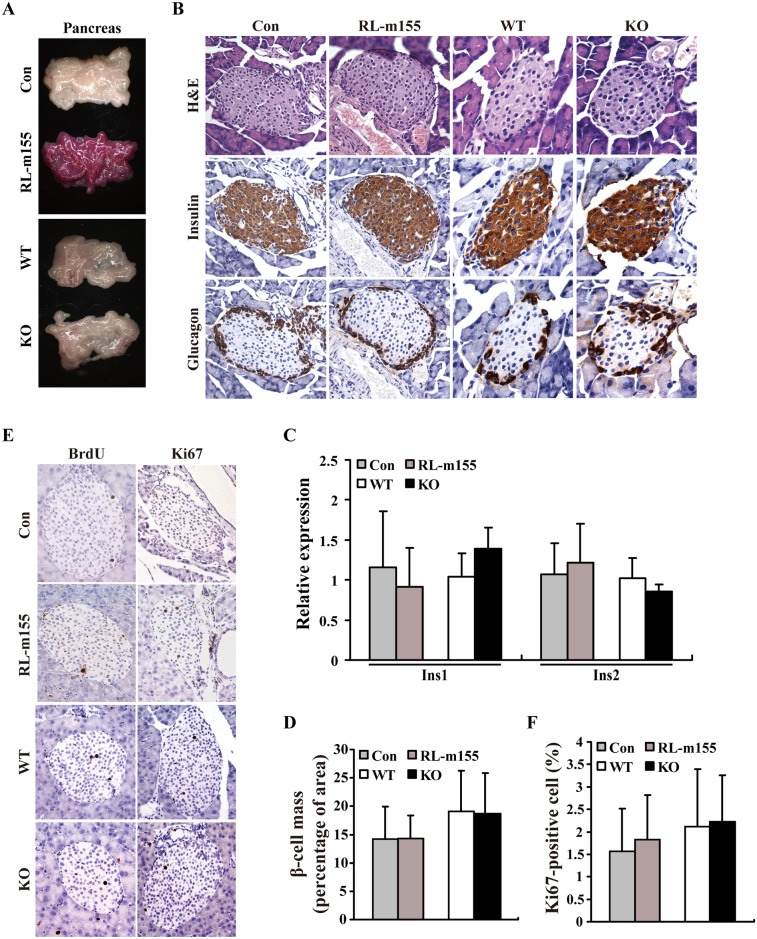
RL-m155 mice and miR-155 knockout (KO) mice displayed the unaltered β-cell proliferation and hormone profiles in pancreas. **(A)** Morphology of the entire pancreas from RL-m155 mice and miR-155 KO mice. **(B)** Haematoxylin and eosin (H&E), insulin and glucagon stainings of pancreatic islets in RL-m155 mice and miR-155 KO mice. **(C)** qRT-PCR analysis of Ins1 and Ins2 mRNA expression in pancreas tissue of RL-m155 mice and miR-155 KO mice. **(D)** Percentage of β-cell mass in RL-m155 mice and miR-155 KO mice. **(E)** BrdU and Ki67 stainings of pancreatic islets in RL-m155 mice and miR-155 KO mice. **(F)** Percentage of Ki67-positive cells in pancreatic islets in RL-m155 mice and miR-155 KO mice. In this study, the non-transgenic littermates/wild-type littermates [i.e., control (con) mice] were used as controls of RL-m155 mice, while wild-type C57BL/6J mice (i.e., WT) of the same age and sex were used as controls of miR-155^–/–^C57BL/6J mice.

**Fig 4:**
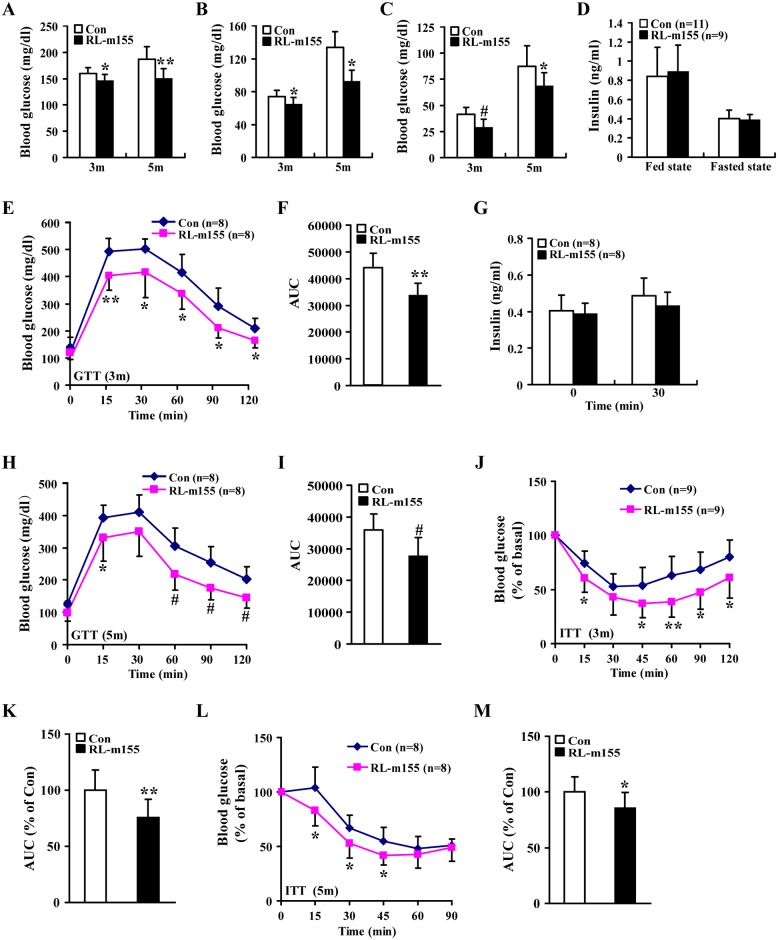
Improved glucose tolerance and insulin sensitivity in RL-m155 mice. **(A-C)** Blood glucose concentrations in fed-state (A), 12-hour–fasted (B) and 24-hour–fasted (C) mice. Control mice: n = 9 (3m) and n = 8 (5m); RL-m155 mice: n = 7 (3m) and n = 9 (5m). **(D)** Serum insulin concentrations in fed-state and 12-hour–fasted mice (5m). **(E-F)** Glucose tolerance test (GTT) in 12-hour–fasted mice (E) and area under the curve (AUC) (F) for this GTT (E). **(G)** Serum insulin measurements performed in 12-hour–fasted mice (3m) during a GTT (E). **(H)** GTT in 12-hour–fasted control (Con) and RL-m155 mice. **(I)** AUC analysis for this GTT (H). **(J)** Insulin tolerance test (ITT) of 12-hour–fasted control and RL-m155 mice. **(K)** AUC analysis for this ITT (J). **(L)** ITT performed on 12-hour–fasted 5-month-old control and RL-m155 mice. **(M)** AUC calculated from mice in (L). Values are statistically significant at *P<0.05; **P<0.01 and ^#^*P*<0.001.

**Fig 5:**
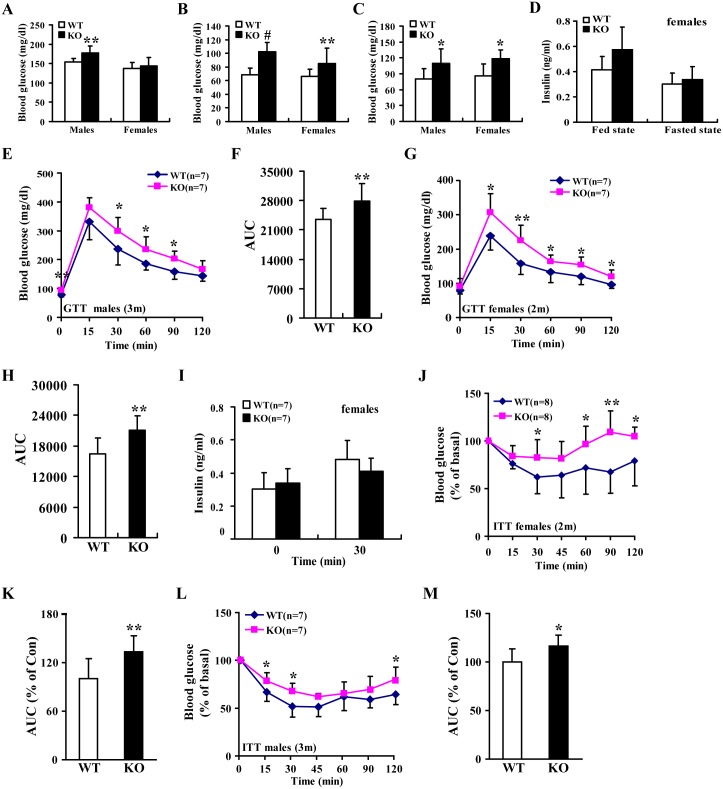
miR-155 deficiency in mice led to hyperglycaemia and insulin resistance. **(A-C)** Blood glucose concentrations in fed-state (A), 12-hour–fasted (B) and 24-hour–fasted (C) miR-155 ^-/-^ mice (males: 3 m and females: 2m). WT mice: n = 10 (males) and n = 12 (females); miR-155 KO mice: n = 9 (males) and n = 10 (females). **(D)** Serum insulin concentrations in fed-state and 12-hour–fasted miR-155 ^-/-^ mice (3m). WT mice: n = 8; miR-155 KO mice: n = 8. **(E-F)** GTT in 12-hour–fasted miR-155 ^-/-^ mice (E) and area under the curve (AUC) (F) for this GTT (E). **(G)** GTT in 12-hour–fasted wild-type (WT) and miR-155 ^-/-^ mice. **(H)** AUC analysis for this GTT (G). **(I)** Serum insulin measurements performed in 12-hour–fasted mice (2m) during a GTT (G). **(J)** ITT of 12-hour–fasted WT and miR-155 ^-/-^ mice. **(K)** AUC analysis for this ITT (J). **(L)** ITT performed on 12-hour–fasted WT and miR-155 ^-/-^ mice. **(M)** AUC calculated from mice in (L). Values are statistically significant at *P<0.05; **P<0.01 and ^#^*P*<0.001.

As mentioned in “Introduction section”, miR-155 might be physiologically required for normal glucose homeostasis. This feature prompted us to firstly explore the influences of miR-155 on pancreatic β-cell proliferation, β-cell mass and β-cell function by using both RL-m155 mice and miR-155 knockout (KO) mice[13]. We found that there was no difference in the morphology of the entire pancreas between control mice (i.e., non-transgenic littermates/wild-type littermates) and RL-m155 mice or between WT mice (i.e., wild-type C57BL/6J mice of the same age and sex) and miR-155 KO mice (Fig 3A). H&E staining and immunostaining of pancreatic sections with antibodies to insulin and to glucagon exhibited normal islet architecture in RL-m155 mice or miR-155 KO mice (Fig 3B). Quantitative analysis also revealed no difference in insulin 1 (Ins1) and insulin 2 (Ins2) expression (Fig 3C), and in total β-cell mass (Fig 3D) between control and RL-m155 mice or between WT and miR-155 KO mice. Moreover, the number of BrdU- and Ki67-positive β-cells did not differ between control and RL-m155 mice or between WT and miR-155 KO mice (Fig 3E and 3F). More importantly, there was also no difference in circulating insulin levels between control and RL-m155 mice (Fig 4D and S3D Fig) or between WT and miR-155 KO mice (Fig 5D), while glucose-stimulated insulin secretion (GSIS) tests revealed the unaltered insulin secretion following a glucose challenge between control and RL-m155 mice (Fig 4G and S3G Fig) or between WT and miR-155 KO mice (Fig 5I). Taken together, our findings support that in mouse pancreas, miR-155 don’t have obvious effects on the pancreatic morphology, β-cell proliferation, β-cell mass and β-cell function.

### Global overexpression of miR-155 improves glucose metabolism and insulin sensitivity in RL-m155 mice

Our above-mentioned results exhibited that the gain and loss of miR-155 function in mice didn’t have influences on pancreas morphology, islet architecture, β-cell proliferation and mass, and insulin and glucagon immunoreactivity (Fig 3), leading us to further explore the roles of miR-155 in normal glucose homeostasis and insulin sensitivity using RL-m155 mice.

We observed that fasting and fed-state blood glucose levels were lower in RL-m155 male (Fig 4A–4C) and female (S3A–S3C Fig) mice, whereas circulating insulin levels in the fed and fasted states did not differ between control and RL-m155 mice (Fig 4D and S3D Fig). Glucose tolerance tests (GTTs) indicated that RL-m155 males and females cleared glucose more efficiently than controls (Fig 4E, 4F, 4H and 4I and S3E and S3F Fig), suggesting an improved glucose tolerance in RL-m155 mice.

**Fig 6:**
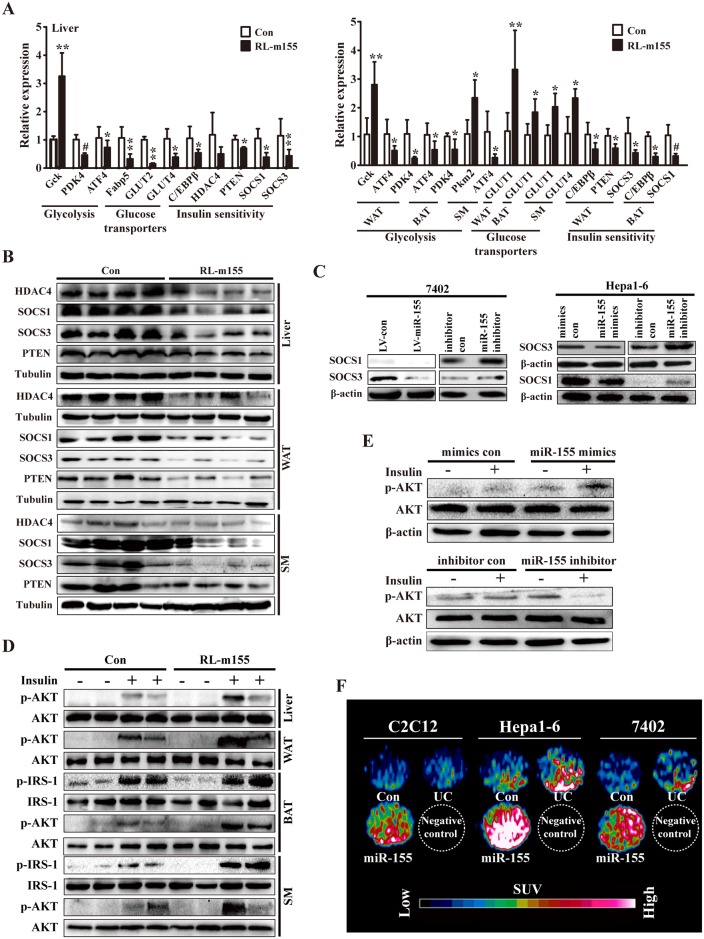
Regulation of gene expression and insulin signalling by miR-155. **(A)** qRT-PCR analysis of the indicated gene expression in liver, WAT, BAT and SM of RL-m155 mice (4m). n = 5–10 mice per indicated genes. Values are statistically significant at *P<0.05; **P<0.01 and ^#^*P*<0.001. **(B)** Western blot analysis of the expression of the indicated proteins in liver, adipose tissue and SM of RL-m155 mice (4m; n = 4). **(C)** Western blot analysis for SOCS1, SOCS3 and HDAC4 expression in miR-155-expressing 7402 cells, 7402 cells transfected with miR-155 inhibitor, and hepa1-6 cells transfected with miR-155 mimics or miR-155 inhibitor. **(D)** Representative Western-blot analysis of insulin-stimulated AKT and IRS-1 (Insulin receptor substrate 1) phosphorylation in liver, adipose tissue or SM of control and RL-m155 mice (5m). **(E)** Representative immunoblot analysis of insulin-stimulated AKT phosphorylation versus total AKT protein levels in hepa1-6 cells transfected with miR-155 mimics or miR-155 inhibitor. **(F)** In vitro evaluation of cellular ^18^F-FDG uptake in miR-155-expressing 7402 cells, and hepa1-6 and C2C12 cells transiently transfected with miR-155 mimics by MicroPET/CT. The data on the fold changes in ^18^F-FDG uptake between miR-155-expressing indicated cells and the corresponding control cells were shown in S9 Fig. UC: untransfected cells.

To evaluate insulin sensitivity of peripheral tissues, we performed insulin tolerance tests (ITTs) in RL-m155 mice. We noticed that RL-m155 mice were more sensitive to insulin than controls (Fig 4J–4M and S3H–S3K Fig). To demonstrate that the lower blood glucose was due to an increase in peripheral tissue insulin sensitivity rather than an increase in secreted insulin, we performed glucose-stimulated insulin secretion (GSIS) tests, and found the unaltered insulin secretion following a glucose challenge between RL-m155 and control mice (Fig 4G and S3G Fig), indicating that improved glucose metabolism in RL-m155 mice results from improved insulin sensitivity of peripheral tissues, but not increased insulin secretion. Taken together, analysis of GSIS, morphological and structure analysis of pancreatic islets, and determination of pancreatic β-cell mass and proliferation revealed no alterations (Figs 3, 4D and 4G, S3D and S3G Fig), further supporting the hypothesis that improved glucose metabolism in RL-m155 mice arises primarily from improved insulin sensitivity of peripheral tissues rather than altered insulin secretion. This increase in insulin sensitivity of peripheral tissues of RL-m155 mice was verified by glucose uptake studies and molecular studies performed in insulin target organs (Fig 6).

Additionally, the glucose metabolic performance in RL-155 mice maintained on an HFD (high-fat diet) was explored in this study. When mice were maintained on the chow diet, the RL-155 mice exhibited lower fasting serum glucose levels than control mice (S4A Fig). The levels of fasting serum glucose were remarkably increased in mice on the HFD (S4A Fig), but the serum glucose levels were significantly decreased in RL-155 mice relative to controls (S4A Fig). When challenged with an i.p. glucose load, RL-155 mice on both chow and HFD displayed significantly improved glucose tolerance (S4B Fig). ITT tests also illustrated improved insulin sensitivity in RL-155 mice on both chow and HFD (S4C Fig). Thus, the global overexpression of miR-155 improves glucose tolerance and whole body insulin sensitivity, even when mice are challenged with a HFD.

### Impaired glucose metabolism in miR-155-deficient mice

To further examine the effects of loss of miR-155 function on blood glucose levels, glucose tolerance and insulin sensitivity, we used miR-155^-/-^ mice to perform the following loss-of-function experiments. miR-155^-/-^ mice exhibited increased blood glucose levels (Fig 5A–5C), and unaltered plasma insulin concentrations (Fig 5D). We observed impaired glucose tolerance (Fig 5E–5H) and insulin resistance (Fig 5J–5M) in miR-155^-/-^ mice, which is contrary to the results of RL-m155 mice. Moreover, GSIS test revealed that serum insulin levels for mice of both genotypes were similar during GTT analysis (Fig 5I), suggesting that impaired glucose metabolism in miR-155^-/-^ mice results from reduced insulin sensitivity of peripheral tissues, but not decreased insulin secretion.

Collectively, analysis of GSIS, morphological and structure analysis of pancreatic islets, and determination of pancreatic β-cell mass and proliferation revealed no alterations (Figs 3, 5D and 5I), further supporting the hypothesis that impaired glucose metabolism in miR-155^-/-^ mice arises primarily from insulin resistance of peripheral tissues rather than impaired insulin secretion. In summary, these observations exhibited that targeted disruption of miR-155 in mice specifically impairs glucose metabolism through induction of insulin resistance.

### miR-155 promotes cellular ^18^F-FDG uptake in 7402, hepa1-6 and C2C12 cells

Diabetes and insulin resistance are associated with defects in glucose uptake, while GTTs and ITTs revealed a positive regulatory role of miR-155 in glucose tolerance and insulin sensitivity, further supporting the hypothesis that miR-155 might drive increased glucose uptake. To better characterize the role of miR-155 in regulating insulin sensitivity in liver and SM, we performed in vitro glucose uptake assays [i.e., ^18^F-FDG (fluoro-D-glucose) uptake assays] using human hepatocellular carcinoma 7402 cells, and murine hepa1-6 and C2C12 cell lines. Here we designed a method for in vitro evaluation of cellular ^18^F-FDG uptake by microPET/CT Inveon scanner, as described previously[14]. ^18^F-FDG microPET/CT scan was employed to reveal that miR-155-expressing 7402 cells, and hepa1-6 and C2C12 cells transiently transfected with miR-155 mimics exhibited significantly enhanced cellular ^18^F-FDG uptake (Fig 6F and S9 Fig), further supporting that miR-155 plays a positive role in regulating insulin sensitivity of peripheral tissues.

### Enhanced glycolysis and insulin-stimulated AKT phosphorylation in peripheral tissues of RL-m155 mice

To further investigate the mechanisms involved in enhanced insulin sensitivity of peripheral tissues in RL-m155 mice, we analyzed various molecules involved in glycolysis, glucose transporters and insulin sensitivity, and insulin-stimulated AKT phosphorylation in peripheral tissues (i.e., liver, adipose tissues and SM) of RL-m155 mice.

We hypothesized that increased glucose uptake induced by miR-155 overexpression (Fig 6F and S9 Fig) represented an increase in glycolytic metabolism. To determine whether glycolysis might be altered in liver, WAT, BAT and SM of RL-m155 mice, we analyzed the expression of several key genes, such as Gck, pyruvate dehydrogenase kinase 4 (PDK4), pyruvate kinase M2 (PKM2) and activating transcription factor 4 (ATF4), involved in this process and whose expression is regulated by insulin in the liver[15]. In RL-m155 mice, Gck expression was increased in liver and WAT, and pyruvate kinase M2 (PKM2) expression was enhanced in SM, while pyruvate dehydrogenase kinase 4 (PDK4) and activating transcription factor 4 (ATF4) expression were reduced in liver, WAT, BAT or SM (Fig 6A and S5 Fig), suggesting that in RL-m155 mice, glycolysis is stimulated in these tissues examined by global miR-155 overexpression. Summarily, consistent with enhanced glucose uptake (Fig 6F and S9 Fig), glucose utilization was promoted in liver, WAT, BAT and SM of RL-m155 mice, as indicated by increased (Gck and PKM2) or decreased (PDK4) expression of enzymes regulating glycolysis. Moreover, in RL-m155 mice, we observed the reduced expression of genes that encode glucose transporters (GLUT) such as GLUT2 and GLUT4 in liver (Fig 6A), suggesting that the decreased expression of GLUT2 and GLUT4 can stop liver from releasing glucose to blood of RL-m155 mice. In contrast, we found the elevated expression of GLUT1 in WAT, BAT and SM, and GLUT4 in SM (Fig 6A), indicating the enhanced absorption of glucose from blood to WAT, BAT and SM of RL-m155 mice. Together, these data suggest that global overexpression of miR-155 alters the metabolic state of these tissues, driving enhanced glucose uptake and favoring glycolytic metabolism.

**Fig 7:**
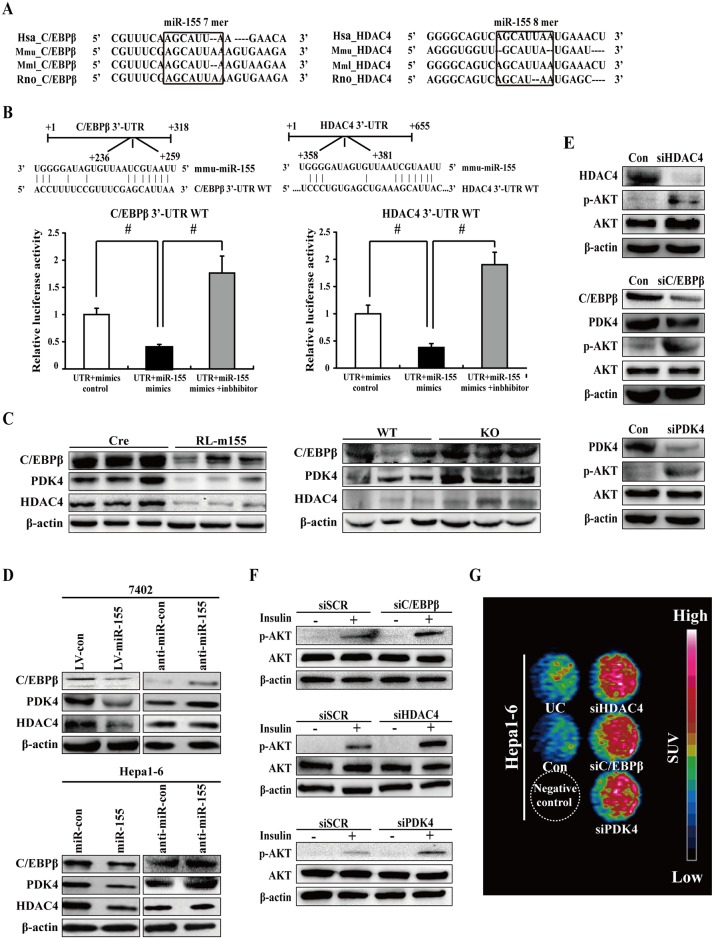
Downregulation of C/EBPβ, HDAC4 and PDK4 in cultured liver cells enhanced insulin-stimulated AKT activation and cellular ^18^F-FDG uptake. **(A)** Sequence alignment of 3’-UTR of human (Hsa), mouse (Mmu), rhesus (Mml) and rat (Rno) C/EBPβ highlighting miR-155 binding site. **(B)** Both C/EBPβ and HDAC4 are target genes of miR-155. The luciferase reporter assay was performed using Hepa1-6 cells as described in the Materials and methods section. Values are statistically significant at ^#^*P*<0.001. **(C)** Immunoblot analysis of C/EBPβ, HDAC4 and PDK4 expression in livers of RL-m155 mice and miR-155 ^-/-^ mice. **(D)** Western blot analysis for C/EBPβ, HDAC4 and PDK4 expression in miR-155-expressing 7402 cells, 7402 cells transfected with miR-155 inhibitor, and hepa1-6 cells transfected with miR-155 mimics or miR-155 inhibitor. **(E)** Cell extracts from hepa1-6 cells transfected with the indicated siRNA oligonucleotides were analyzed by immunoblotting with antibodies against the indicated proteins. **(F)** Western blot analysis of insulin-stimulated phosphorylation of AKT in hepa1-6 cells transfected with the indicated siRNA oligonucleotides. **(G)** In vitro evaluation of cellular ^18^F-FDG uptake in hepa1-6 cells transfected with the indicated siRNA oligonucleotides by MicroPET/CT. The data on the fold changes in ^18^F-FDG uptake between siRNA-transfected hepa1-6 cells and the corresponding control cells were shown in S10 Fig. UC: untransfected cells

Next, we analyzed the expression of known negative regulators (i.e., C/EBPβ[16], HDAC4[17] and PTEN[18]) of insulin sensitivity and known inducers (i.e., SOCS1 and SOCS3[19, 20]) of insulin resistance. As expected, the global overexpression of miR-155 in mice reduced the expression of C/EBPβ, HDAC4, PTEN, SOCS1 and SOCS3 in liver, adipose tissue and SM of RL-m155 mice (Figs 6A, 6B and 7C), while our results from 7402 and hepa1-6 cells revealed that miR-155 negatively regulated the expression of C/EBPβ, HDAC4, SOCS1 and SOCS3 (Figs 6C and 7D), indicating that decreased expression of C/EBPβ, HDAC4, PTEN, SOCS1 and SOCS3 in insulin target organs of RL-m155 mice is consistent with increased whole-body insulin sensitivity.

The metabolic effects of insulin, including insulin sensitivity, glucose uptake and glycogen synthesis, are mediated through activation of PI3K-AKT signaling pathway[21]. The phosphorylation of AKT and IRS-1 (Insulin receptor substrate 1) was enhanced in liver, adipose tissue or SM of RL-m155 mice following stimulation by insulin (Fig 6D). At a molecular level, improved insulin sensitivity in RL-m155 mice (Fig 4 and S3 Fig) was paralleled by increased insulin-stimulated phosphorylation of AKT and IRS-1 in liver, adipose tissue or SM (Fig 6D). Together global miR-155 overexpression in mice enhances insulin sensitivity in liver, muscle and fat cells of RL-m155 mice.

Furthermore, Western-blot analysis revealed that insulin-stimulated AKT phosphorylation was increased in hepa1-6 cells transfected with miR-155 mimics (Fig 6E), whereas insulin-stimulated AKT phosphorylation was reduced in hepa1-6 cells transfected with miR-155 inhibitor (Fig 6E), suggesting that miR-155 is a positive regulator of insulin sensitivity. Summarily, our results indicate that miR-155 affects insulin's ability to positively regulate AKT phosphorylation in liver cells.

### Silencing of miR-155 targets (i.e. C/EBPβ and HDAC4) and C/EBPβ target PDK4 in cultured liver cells enhanced insulin-stimulated AKT activation and glucose uptake

Subsequently, we want to address the possible mechanisms by which miR-155 regulates insulin sensitivity and glucose metabolism. The putative or verified miR-155 target genes are summarized in S5 Table. Among miR-155 target genes, C/EBPβ, HDAC4 and SOCS1 especially caught our attention. The reasons are as follows: (1) C/EBPβ, HDAC4 and SOCS1 genes harbor miR-155 binding site, which is conserved across different phyla (Fig 7A and S7A Fig); (2) C/EBPβ[16, 22], HDAC4[23, 24] and SOCS1[25] are negative regulators of blood glucose and insulin sensitivity in mice, and SOCS1 is characterized as a positive mediator of insulin resistance[25]; (3) SOCS1 is a negative regulator of IRS-1/PI3K/AKT insulin pathway[26, 27].

The 3’-UTRs of C/EBPβ (Fig 7B), HDAC4 (Fig 7B) and SOCA1 (S7B Fig) mRNA contain complementary site for the seed region of miR-155, respectively. We generated reporter constructs in which the luciferase coding sequence was fused to the 3′-UTRs of these genes. Measurements of luciferase activity in hepa1-6 cells transfected with miR-155 mimics plus reporter plasmid containing the wild-type 3’-UTRs of C/EBPβ or HDAC4 exhibited a significant reduction of luciferase activity (Fig 7B), respectively. In contrast, transient transfection of wild-type C/EBPβ-Luc reporter or HDAC4-Luc reporter with miR-155 mimics plus inhibitor into hepa1-6 cells could fully reverse the miR-155-induced decrease in luciferase activity (Fig 7B).

To examine the influences of miR-155 on endogenous expression of these miR-155 targets, we firstly determined their expression in the livers of RL-m155 mice and miR-155^-/-^ mice. As expected, mRNA and protein levels of C/EBPβ, HDAC4 and SOCS1, and C/EBPβ target PDK4 in the liver of RL-m155 mice was remarkably down-regulated (Figs 6A, 6B and 7C, S5 and S7C Figs), whereas C/EBPβ, HDAC4, SOCS1 and PDK4 protein levels in the livers of miR-155^-/-^ mice were significantly up-regulated (Fig 7C and S7C Fig). Moreover, miR-155 overexpression in 7402 and hepa1-6 cells resulted in significant reduction of endogenous C/EBPβ, HDAC4, SOCS1 and PDK4 (Fig 6C and S8 Fig). Conversely, 7402 and hepa1-6 cells transfected with miR-155 inhibitor exhibited the enhanced expression of C/EBPβ, HDAC4, SOCS1 and PDK4 (Fig 6C and S8 Fig). Therefore, miR-155 negatively regulates it targets C/EBPβ, HDAC4 and SOCS1 expression, and C/EBPβ target PDK4 expression. More importantly, these aforementioned results suggest that C/EBPβ and HDAC4 are direct targets of miR-155. Furthermore, SOCS1 is identified as a direct target gene of miR-155 in human and mouse cells[28–30].

To directly examine potential roles for C/EBPβ and HDAC4, and C/EBPβ target PDK4 in control of insulin-stimulated AKT activation and glucose uptake, we analyzed AKT phosphorylation and glucose uptake in insulin-stimulated hepa1-6 cells transfected with short interfering RNAs (siRNAs) directed to mouse C/EBPβ (siC/EBPβ), HDAC4 (siHDAC4) and PDK4 (siPDK4), respectively. Western-blot and qRT-PCR analysis showed successful reduction of C/EBPβ, HDAC4 and PDK4 expression in cells transfected with siC/EBPβ, siHDAC4 and siPDK4, respectively (Fig 7E and S8 Fig). siRNA-mediated silencing of C/EBPβ, HDAC4 or PDK4 in hepa1-6 cells enhanced insulin-stimulated AKT phosphorylation (Fig 7F), respectively, similar to what was observed upon miR-155 overexpression (Fig 6E). Taken together, our results indicate that C/EBPβ, HDAC4 or PDK4 affects insulin's ability to regulate AKT phosphorylation in liver cells. More importantly, our findings suggest that C/EBPβ and HDAC4 are involved in miR-155-mediated insulin-stimulated AKT phosphorylation in liver cells. Moreover, siRNA-mediated knockdown of miR-155 target genes (C/EBPβ and HDAC4) and C/EBPβ target gene PDK4 mimicked miR-155-induced glucose uptake in hepa1-6 cells (Fig 7G and S10 Fig), which is similar as the results caused by miR-155 overexpression (Fig 6F and S9 Fig), suggesting that C/EBPβ and HDAC4 are involved in miR-155-induced glucose uptake.

## Discussion

Altered expression of miRNAs in insulin-sensitive tissues of T2D patients suggests a potential role for these small RNA molecules in the complications associated with the diabetic condition[1, 3, 31]. Moreover, increasing evidence has revealed that miRNAs are also present in a stable form in several body fluids, including blood, suggesting that extracellular miRNAs hold promise to serve as novel biomarkers for metabolic disorders and/or their associated complications[1, 3, 31]. To date, a number of studies have revealed an altered profile of circulating miRNAs in various metabolic diseases such as T2D[1, 3, 31]. miR-155 expression in PBMCs from T2D patients was decreased[11], which is consistent with our findings that the downregulated miR-155 levels were found in serum from T2D patients. Moreover, circulating levels of miR-155 were downregulated in plasma from patients with coronary artery disease plus diabetes[32], while miR-155 expression was reduced in diabetic kidney, heart, aorta, PBMCs and sciatic nerve of diabetic rats[33]. But the causal relationship between miR-155 dysregulation and diabetes or diabetes complications remains unknown, and further investigations are needed to precisely clarify the roles of miR-155 in diabetes and diabetes complications, and the underlying mechanisms.

Moreover, it is hard to exactly tell where the serum miRNAs are from because it is difficult to get biopsy samples to identify which specific tissue changes blood miRNA levels in these T2D patients. It is well known that animal and human living tissues can release the intracellular miRNAs into the circulation[1, 3, 31]. When human tumors were implanted in mice, specific human tumor-derived miRNAs have been detected in plasma[34], while the circulating myocardial-derived miRNAs might be useful as potential biomarkers for infarction[35–39]. Indeed, circulating miRNAs can provide an integrated view of the metabolic profile of the T2D patients because all insulin-sensitive tissues release the packaged miRNA into blood[1, 3, 31]. In the T2DM rat model (obese high fat diet animals treated with streptozotocin), the rat blood miRNA profile was clustered closely to those from the rat insulin-sensitive tissues (skeletal muscle, adipose tissue and liver) and pancreas[40]. Additionally, most of the miRNA changes detected in these tissues involved in the insulin signaling pathway were also detected in blood miRNA profile[40]. Thus, we suspect that the decrease of miR-155 expression in insulin-sensitive tissues (i.e., liver, adipose tissue and skeletal muscle) may cause the decreased blood miR-155 level in diabetic patients. In future study, we can prove it by observing the changes of miR-155 expression in different insulin-sensitive tissues and blood during the development of diet-induced obesity and diabetes in mice.

In this study, when globally overexpressed in mice, miR-155 resulted in hypoglycaemia, improved glucose tolerance and enhanced insulin sensitivity of peripheral tissues, whereas mice lacking miR-155 developed hyperglycemia, glucose intolerance and insulin resistance, suggesting the beneficially regulatory roles of miR-155 in glucose homeostasis. Increased insulin sensitivity and improved glucose tolerance in RL-m155 mice could be explained, at least in part, by enhanced glucose uptake through elevated phosphorylation of AKT, and by enhanced glycolysis manifested via upregulation of Gck and PKM2, and downregulation of PDK4. All in all, our findings firstly demonstrate that miR-155 regulates multiple aspects of glucose metabolism. More importantly, our findings fully reveal that the gain of miR-155 function leads to hypoglycemia and improved glucose tolerance through induction of insulin insensitivity in peripheral tissues, thereby improving whole-body glucose metabolism.

Up to now, there have been very few miRNAs, such as miR-130a-3p[41], miR-26a[42] and miR-155 (this study), to be found to act as positive regulators of glucose tolerance and insulin sensitivity in vivo. Interestingly, our study revealed that miR-155 overexpression in mice fed a conventional diet resulted in the aforementioned multiple metabolic phenotypes, whereas miR-26a overexpression led to reduced blood-glucose levels, better glucose tolerance and insulin sensitivity, and decreased hepatic glucose production in high-fat diet-fed mice, but not in conventional diet–fed mice[42], suggesting that miR-155 and miR-26a might play different roles in regulating glucose metabolism.

**Fig 8:**
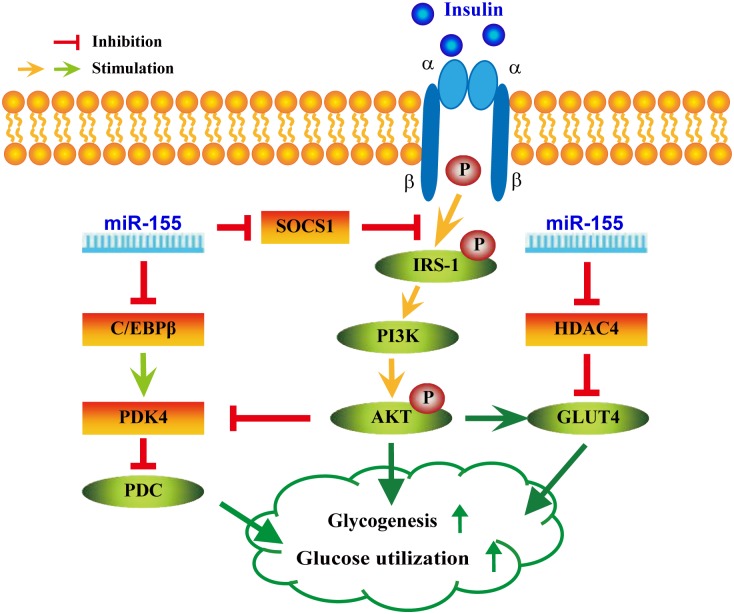
A proposed model on the positive roles of miR-155 in glucose metabolism by coordinated regulation of multiple genes.

As shown in Fig 8 and S11 Fig, this study uncovers partially molecular mechanisms underlying miR-155’s functions in the above-mentioned multiple metabolic phenotypes. miRNAs are critical modulators of glucose and lipid metabolism by negatively regulating the expression of multiple target genes[1, 3]. Our studies revealed that the expression of miR-155 targets (i.e. C/EBPβ, HDAC4 and SOCS1) and PDK4, a direct target of C/EBPβ[43, 44], were negatively regulated by miR-155, and C/EBPβ knockdown reduced PDK4 expression in hepa1-6 cells. Both C/EBPβ^–/–^mice[16, 22] and PDK4^–/–^mice[45, 46] display hypoglycemia and increased insulin sensitivity. PDK4 plays a crucial role in glucose utilization by negatively regulating pyruvate dehydrogenase complex (PDC) activity[43, 44]. Furthermore, the activation of AKT signaling by insulin suppresses PDK4 expression[43, 44]. These aforementioned data indicate that the metabolic phenotypes in mice with loss of function of C/EBPβ[16, 22] or PDK4[45, 46] are similar to what was observed upon miR-155 overexpression in mice, and are opposite to what we found in miR-155^-/-^ mice. Therefore, these observations support that miR-155 might negatively regulate PDK4 via negatively modulating C/EBPβ expression, thereby resulting in the aforementioned metabolic phenotypes (Fig 8 and S11 Fig).

Defects in peripheral tissue glucose uptake are associated with insulin resistance. We found that miR-155 overexpression decreased miR-155 target gene HDAC4 expression and increased GLUT4 levels in SM of RL-m155 mice, and increased glucose uptake in cells (including C2C12 cells) examined, similar to what was observed upon HDAC4 siRNA. HDAC4 inhibits the expression of GLUT4, a direct target of HDAC4[47]. HDAC4 siRNA increased GLUT4 levels and glucose uptake in adipocytes[48]. Loss of the class IIa HDACs (HDAC4,5, and 7) in murine liver resulted in lowered blood glucose levels, increased hepatic glycogen storage, improved glucose tolerance and insulin sensitivity in mice[23, 24]. Together, these data support this hypothesis that miR-155 upregulates GLUT4 expression through downregulating HDAC4 expression, thereby leading to enhanced glucose uptake in insulin-sensitive tissues (i.e., SM), strongly supporting that miR-155 play a positive role in regulating insulin sensitivity of peripheral tissues, at least in part, through suppressing HDAC4 expression (Fig 8).

SOCS1, a negative regulator of blood glucose and insulin sensitivity in mice[19, 20, 25], blocks IRS-1/PI3K/AKT insulin pathway by ubiquitin-mediated degradation of IRS-1[26, 27].

Our results revealed that miR-155 negatively regulated its target gene SOCS1 expression in insulin-sensitive tissues and liver cells, and miR-155 overexpression led to enhanced IRS-1 phosphorylation and AKT phosphorylation in SM and adipose tissue of RL-m155 mice after insulin treatment. Collectively, the findings prompt us to speculate that miR-155 might activate IRS-1/PI3K/AKT insulin pathway by inhibiting SOCS1 expression, which at least partially contributes to the above-mentioned metabolic phenotypes (Fig 8 and S11 Fig).

As shown in Fig 8 and S11 Fig, here we provide a working hypothesis that explains the roles of miR-155 in inducing multiple phenotypic changes in mice and the underlying mechanisms. Although we cannot rule out the possibility that other known (such as CES3)[8] and unknown target genes of miR-155 (S5 Table) contribute to glucose metabolism, we speculate that the coordinated regulation of the miR-155 target genes (C/EBPβ, HDAC4 and SOCS1) could profoundly alter gene expression profiling related with glucose metabolism, thereby modulating the above-mentioned multiple metabolic phenotypes. These results from this study and the recent report[42] demonstrate that a single miRNA, such as miR-155 (this study) and miR-26a[42], can regulate multiple metabolic phenotypes in vivo by coordinated regulation of multiple genes. These effects of miR-155 could be greatly consolidated and augmented by crosstalk between glucose metabolism and insulin signaling, which indicates that a small change in miR-155 expression can sometimes have a large physiological effect on metabolic phenotypes. In line with this idea, a minor or modest increase in miR-155 expression in mice was sufficient to induce multiple phenotypic changes.

Besides these aforementioned miR-155 targets, many metabolic genes that lack predicted miR-155 target sites exhibit the altered expression upon modulation of miR-155 expression.

These glucose metabolism-related genes are involved in glycogen metabolism (Gys2), glycolysis (Gck, PDK4, PKM2 and Ldha), glucose transporters (GLUT1, GLUT2, GLUT4, Slc1a2 and Slc3a2). It is likely that these differentially expressed genes act the downstream of target genes of miR-155. Indeed, as mentioned above, PDK4[43, 44] is C/EBPβ target gene, and GLUT4[47] is a target gene of HDAC4.

Furthermore, there are several lines of evidence that miR-155 is also involved in adipocyte differentiation[49], adipogenesis[50, 51] and lipid metabolism[8, 52]. In vivo, miR-155 overexpression in transgenic mice caused the reduction of brown adipose tissue mass and impairment of brown adipose tissue function, whereas, miR-155 inhibition in mice resulted in a hyperactive brown adipose tissue and induced a brown adipocyte-like phenotype ('browning') in white adipocytes[49]. The ectopic expression of miR-155 significantly inhibited adipogenesis in vitro[50, 51]. Our previous report revealed that liver-specific overexpression of miR-155 transgene resulted in significantly reduced levels of serum total cholesterol, triglycerides (TG) and high-density lipoprotein (HDL), as well as remarkably decreased contents of hepatic lipid, TG, HDL and free fatty acid in Rm155LG/ Cre transgenic mice[8], indicating that miR-155 negatively modulates levels of hepatic and serum lipid compositions, and displays lipid-lowering activity in mice. In summary, these findings from this study and other studies demonstrate the importance of miR-155 in the multiple aspects of glucose metabolism and insulin signaling, lipid metabolism and adipocyte differentiation through regulation of critical metabolic genes.

Furthermore, these findings from this study and other studies revealed the decreased BAT mass and function in miR-155 transgenic mice, and the preliminary data from this study displayed the enhanced glucose uptake & glycolysis and insulin-stimulated AKT phosphorylation in BAT of RL-m155 mice. But the influences of the decreased BAT mass and function caused by miR-155 overexpression in mice on glucose metabolism (including whole-body insulin sensitivity) in mice remains unknown. In future study, given the important roles of BAT in obesity and glucose metabolism, the conditional gain-of-function (using Rm155LG transgenic mice[8]) and loss-of-function (using miR-155 floxed mice[53]) of miR-155 in BAT of mice will be employed to fully explore the roles of the miR-155 overexpression-induced decreased BAT mass and function on glucose metabolism (including whole-body insulin sensitivity) in mice.

Additionally, further experiments in primates will be required to evaluate the roles of miR-155 in improving glucose tolerance and insulin sensitivity, and subsequently lowering blood glucose levels, which will be helpful investigations in assessing the prospects for therapeutical miR-155 gain of function by using miR-155 mimics to treat insulin resistance and T2DM.

Besides the multifunctions of miR-155, it is important to pay attention to the following aspects. First, miR-155 regulates multiple components of glucose metabolism. Second, our results from this study and previous report[8] reveal the blood glucose-lowering activity and lipid-lowering activity of miR-155. Third, miR-155 transgene is universally expressed at relatively low levels in liver, WAT, BAT, SM, pancreas and isolated islets of RL-m155 mice, and in liver of Rm155LG/Alb-Cre mice[8], while miR-155 transgene is expressed at relatively high levels in brain, testis and heart of RL-m155 mice. But our results indicate that the relatively low levels of miR-155 transgene expression in major insulin target organs or tissues (liver, adipose tissues and SM) are sufficient to induce phenotypes described above and observed in our paper[8], indicating that modest overexpression of miR-155 could be safe; in support of this idea, physiological and pathological side effects of miR-155 overexpression were not observed in RL-m155 mice and Rm155LG/Alb-Cre mice of up to one years of age. Thus, the low and modest miR-155 overexpression can decrease the risk of tumorigenesis. Four, the global (this study) or hepatocyte-specific[8] overexpression of miR-155 in mice does not induce weight gain, avoiding the major side effect of increasing insulin sensitivity for diabetes therapies[54]. Given the potent roles of miR-155 in the above observations as well as lipid metabolism[8, 52], these findings suggest miR-155 as a promising novel target for treatment of T2DM.

In summary, our findings firstly reveal that (1) miR-155 regulates multiple aspects of normal glucose metabolism and insulin signaling through coordinated regulation of critical metabolic genes in mice; (2) miR-155 is a positive regulator of insulin sensitivity; and (3) miR-155 is physiologically required for normal blood glucose homeostasis in mice. In addition, miR-155 displays blood glucose-lowering activity and lipid-lowering activity in mice. More importantly, our observations strongly support that miR-155 is entitled to control all 3 hallmarks of T2DM, namely insulin resistance, excessive HGP which primarily results from sustained gluconeogenesis[55], and elevated lipid synthesis. Although the mechanism(s) of how miR-155 controls glucose homeostasis clearly requires further investigation, these current findings suggest that the therapeutical gain of miR-155 function may become a beneficial strategy for glycemic control to treat insulin resistance and T2DM. Summarily, miR-155 represents a promising target for the treatment of insulin resistance and diabetes.

## Materials and Methods

### Animal studies with miR-155 transgenic mice and miR-155 ^-/-^ mice

The homozygous EIIa-Cre transgenic mice (FVB/N-Tg(EIIa-cre)C5379Lmgd/J) and the wild-type FVB/N mice were obtained from Model Animal Research Center of Nanjing University. The wildtype C57BL/6J mice were purchased from Center of Experimental Animals, Southern Medical University.

Rm155LG transgenic mice that can conditionally overexpress mouse miR-155 transgene mediated by Cre/lox P system have been generated on C57BL/6 background, as previously described[8]. RL-m155 transgenic mice have been generated on FVB background, as described in S1 and S2 Figs miR-155^–/–^C57BL/6J mice (B6.Cg-Mir155^tm1.1Rsky^/J; Stock Number: 007745) were purchased from Jackson laboratory[13].

This study was approved by the Southern Medical University (approval number: L2015079) and was carried out in strict accordance with the recommendations in the Guide for the Care and Use of Laboratory Animals of the Southern Medical University. The protocol was approved by the Committee on the Ethics of Animal Experiments of the Southern Medical University. All surgery was performed under sodium pentobarbital anesthesia, all efforts were made to minimize animal suffering and the number of animals used was kept to a minimum by the experimental design.

### Patients and healthy subjects

Newly diagnosed T2DM patients (n = 30) and healthy controls (n = 30) were recruited at Department of Endocrinology, the Second Affiliated Hospital of Guangzhou Medical University. Patients in this study were diagnosed according to the World Health Organization (WHO) diagnostic criteria[56]. To minimize the likelihood of misclassification of autoimmune diabetes as T2DM, the subjects with any of four auto-antibodies (GADA, IA-2A, ICA, or IAA) were excluded from T2DM. Non-diabetes subjects as control were healthy subjects without any family history of diabetes, and with fasting plasma glucose levels below 6.1 mM and 2 h post-load plasma glucose concentrations <7.8 mM after a 75 g oral glucose tolerance test. The present study was approved by the Hospital Ethics Committee of the Second Affiliated Hospital of Guangzhou Medical University (Project No. 015027).

Insulin resistance (HOMA-IR) and β-cell function (HOMA-β) as indicators of homeostasis model assessment were utilized to assess the status of insulin action and insulin secretion in subjects, respectively. HOMA-IR = FPG (mmol/l)* FINS (mU/ml)/22.5, HOMA-β = 20* FINS (mU/ml)/[FPG (mmol/l)-3.5][57].

### Quantitative RT-PCR (qRT-PCR)

To quantitate miRNA and mRNA expression, total RNA was extracted from cells or from various tissues of transgenic mice and gene knockout mice with the use of TRIzol reagent (TaKaRa). The isolation of microRNA from the serum of healthy subjects and T2D patients was performed using the microRNA RNeasy Mini Kit (Exiqon) following the manufacturer’s recommendations. qRT-PCR was performed with standard methods on Stratagene Mx3005P qRT-PCR System, which are described in detail previously[8]. The primers used for the amplification of the indicated genes were listed in S1–S3 Tables.

### Organ and tissue fluorescence imaging

mRFP expression in the postnatal organs and tissues of RL-m155 transgenic mice was assayed under stereo fluorescent microscope (Nikon, AZ100), as described previously[8, 58, 59].

### Ex vivo organ optical imaging of firefly luciferase (Luc) activity

Bioluminescence imaging for multiple organs and tissues obtained from RL-m155 transgenic and control mice was measured using the IVIS LuminaIIimaging system (Xenogen Corp., Alameda, CA), as described previously[8, 58].

### Metabolic studies

For glucose tolerance test (GTT), mice were fasted overnight for 12h, and then injected intraperitoneally with glucose (2g/kg body weight). For insulin tolerance test (ITT), mice were fasted overnight for 6h, and then injected intraperitoneally with human insulin (Novo Nordisk) at a dose of 0.75IU/kg body weight. Blood glucose levels were measured from mouse tail vein with an automatic glucometer (One Touch Lifescan, Johnson & Johnson, USA) before glucose and insulin injection and at the indicated time after injection. Serum insulin levels were measured by enzyme-linked immunosorbent assay according to vendor’s instructions (Millipore Rat/Mouse Insulin ELISA Kit, EZRMI-13K).

### Histological and immunohistological examinations

For immunohistology, mouse tissues were fixed in 4% paraformaldehyde at 4°C overnight and embedded in paraffin as described previously[60–62], then followed by hematoxylin and eosin staining (H&E staining) according to standard procedures. Afterward, the sections or slides were stained with immunohistochemistry (IHC). The primary antibodies are anti-insulin [1:100, Cell Signaling Technology (CST), USA], anti-glucagon (1:100; CST, USA), anti-Ki67 (1:300; Abcam) and anti-BrdU (1:50; GE Healthcare).

### Western blot analysis

Western blot was performed with standard methods, which are described in detail previously[60–64]. For origin and description of all antibodies used in this study, see S4 Table.

### miRNAs transient transfection

The mouse or human miR-155 mimics, mimics control, miR-155 inhibitor and inhibitor control were purchased from RiboBio Co., Ltd (Guangzhou, China). miRNAs were transiently transfected into cells (including hepa1-6, 7402 and C2C12 cells) at a working concentration of 100nM using Lipofectamine 2000 reagent (Invitrogen) in accordance with the manufacturer’s procedure. All RNA oligonucleotides treatments proceeded for 48-72h. The effect of in vitro overexpression of miR-155 by using miRNA mimics and the repression of endogenous miR-155 expression by a miR-155 inhibitor on insulin-stimulated AKT phopshorylation was examined in hepa1-6 cells. After stimulation with 50IU/L human insulin (Novo Nordisk) for 15 min at 37°C (S6 Fig), the medium was removed and the cells were immediately lysed with ice-cold lysis buffer.

### Dual luciferase reporter assay

Mouse hepatoma (Hepa1-6) cells were obtained from the Cell Bank of Chinese Academy of Sciences Shanghai Institute of Cell Biology. Hepa1-6 cells were seeded in triplicate in 96-well plates and cultured overnight. The dual luciferase reporter gene plasmids [pLuc-C/EBPβ-3’-UTR-wt or pLuc-HDAC4-3’-UTR-wt (Kangbio, Shenzhen, China)] were cotransfected into hepa1-6 cells with the miR-155 mimics, mimics control or miR-155 mimics plus miR-155 inhibitor using Lipofectamine 2000 Reagent (Invitrogen), respectively. Luciferase and Renilla activities were assayed 48 hours after transfection using the Dual Luciferase Reporter Assay Kit (Promega) following the manufacturer’s instructions.

### RNA interference experiments

An siRNA targeting mouse PDK4 (siG140711153320), an siRNA targeting mouse C/EBPβ (siG09723100032), an siRNA targeting mouse HDAC4 (siB1271190307) and a control siRNA were purchased from RiboBio Co., Ltd (Guangzhou, China). Western-blot and qRT-PCR analysis showed successful reduction of C/EBPβ, HDAC4 and PDK4 expression in cells transfected with siC/EBPβ, siHDAC4 and siPDK4, respectively (Fig 7E and S8 Fig). Hepa1-6 cells were transfected with 50nM siRNA-PDK4, siRNA-C/EBPβ, siRNA-HDAC4 or scrambled control siRNAs using Lipofectamine 2000 (Invitrogen) for 48-72h according to the manufacturer's instructions. The effect of PDK4, C/EBPβ or HDAC4 knockdown on insulin-stimulated AKT phopshorylation was examined in hepa1-6 cells. After stimulation with 50IU/L human insulin (Novo Nordisk) for 15 min at 37°C (S6 Fig), the medium was removed and the cells were immediately lysed with ice-cold lysis buffer for western-blot.

### Cellular ^18^F-FDG uptake

Cells were seeded on a 24-well plate (2×10^5^/well) for microPET/CT scan. After 24 h, culture medium was replaced, and cells were incubated in fresh medium containing ^18^F-FDG (about 20μCi/well) for 1h. Then, cells were washed with phosphate-buffered saline five times, and image was obtained by microPET/CT Inveon scanner (Siemens).

### Statistical analysis

All data were presented as mean±SD. Statistical analysis was performed using a SPSS 13.0 software package. Values are statistically significant at *P<0.05; **P<0.01 and ^#^*P*<0.001.

## Supporting Information

S1 Fig Generation of RL-m155 transgenic mice. **(A)** Schematic strategy for generating RL-m155 transgenic mice. Procedure for producing transgenic mice which can globally overexpress mouse miR-155 transgene in multiple organs and tissues of mice was detailedly illustrated in S1A Fig. Briefly, at 6–8 wk of age, the heterozygous Rm155LG transgenic mice[8] were mated with the homozygous EIIa-Cre transgenic mice (FVB/N-Tg(EIIa-cre)C5379Lmgd/J)[65] to generate F1 (S1B Fig); next, both luciferase (Luc)- and mRFP-positive F1 animals (2^#^, 4^#^ or 6^#^) (shown in S1B-b,c Fig) were intercrossed to produce F2 (shown in S1C Fig), including Luc- and mRFP-positive F2 animals with white fur (6^#^). Finally, both Luc- and mRFP-positive F2 animals with white fur and without Cre gene (determined by PCR-based genotyping) were intercrossed to produce RL-m155 transgenic mice (R: mRFP; L: Luc) (S1D Fig) which can globally and constantly express mRFP (S2A Fig), Luc (S2A Fig) and miR-155 (S2B Fig) transgenes in multiple organs and tissues of RL-m155 transgenic mice. Additionally, the genetic background of RL-m155 mice is FVB/N strain. **(B)** Whole-body fluorescence (b) and bioluminescence (c) imaging for newborn offspring derived from mating heterozygous Rm155LG transgenic mice with homozygous EIIa-Cre mice. **(C)** In vivo mRFP (b) and luc (c) imaging for newborn offspring derived from intercrossing of both Luc- and mRFP-positive F1 animals (i.e., 2^#^, 4^#^ or 6^#^) (shown in S1B-b,c Fig). **(D)** Whole-body fluorescence (b) and bioluminescence (c) imaging for newborn RL-m155 transgenic mice. Both Luc- and mRFP-positive mice (i.e., 4^#^, 5^#^ and 6^#^) (shown in S1D Fig) are RL-m155 transgenic mice. **(E)** Whole-body fluorescence (b) and bioluminescence (c) imaging for adult RL-m155 transgenic mice. **(F)** PCR-based genotyping for Cre, mRFP and Luc transgenes in RL-m155 transgenic mice. These RL-m155 transgenic mice (shown in S1D Fig) were individually analyzed by PCR for the genomic integration of Cre, mRFP and Luc transgenes with tail biopsy-derived DNA. PCR products were amplified by the primer pair P1/P2 (specific for mRFP), by the primer pair P3/P4 (specific for Luc) and by the primer pair P5/P6 (specific for Cre), respectively. P1: 5'-GGGAGCGCGTGATGAAC-3', P2: 5'-CGTTGTGGGAGGTGATGTC-3';P3: 5'-AGATACGCCCT GGTTCCTGG-3',P4: 5'-ACGAACACCACGGTAGGCTG-3';P5: 5'-GAACCTGATGGACATGTTC AGG-3', P6:5'-AGTGCGTTCGAACGCTAGAGCCTGT-3'. lane 1: Cre positive control DNA from EIIa-Cre mouse as template; lane 2: positive control (pRm155LG as template); lane 9: negative control using genomic DNA from WT mouse as template. Data are representative of three independent PCR experiments that yield similar results.
(TIF)

S2 Fig Global overexpression of mouse miR-155 transgene in multiple organs and tissues of RL-m155 transgenic mice. **(A)** mRFP and Luc expression in multiple organs and tissues of RL-m155 transgenic mice. The left organ samples in each figure were obtained from one control littermate, while the right organ samples in each figure were isolated from one RL-m155 transgenic mouse. mRFP expression in the postnatal organs and tissues of RL-m155 transgenic mice was assayed under stereo fluorescent microscope (Nikon, AZ100), while bioluminescence imaging for multiple organs and tissues obtained from RL-m155 transgenic mouse and littermate controls was measured noninvasively using the IVIS LuminaIIimaging system (Xenogen Corp., Alameda, CA). Muscle and pancreas from RL-m155R transgenic mice (the right samples in each figure) can be distinguished from their wildtype littermates according to their deep red color under daylight (Fig 2A). **(B)** qRT-PCR analysis of the expression of miR-155 transgene in multiple organs and tissues of RL-m155 transgenic mice. BAT: brown adipose tissue; WAT: white adipose tissue. (**C**) RL-m155 (right) and control (left) male littermates at 20 weeks of age. (**D**) RL-m155 (right) and control (left) female littermates at age 12 weeks. **(E-F)** Body weight of control mice vs. RL-m155 transgenic mice at different ages. Values are mean ± SD; n = 5–10 mice per time point. *, *P* < 0.05 compared with control mice;**, *P*<0.01 compared with control mice;#, *P*<0.001 compared with control mice.
(TIF)

S3 Fig RL-m155 female mice display improved glucose tolerance and enhanced insulin sensitivity. **(A-C)** Blood glucose concentrations in fed-state (A), 12-hour–fasted (B) and 24-hour–fasted (C) mice at indicated ages. **(D)** Serum insulin concentrations in fed-state and 12-hour–fasted mice (5m). **(E-F)** GTT in 12-hour–fasted mice (E) and AUC (F) for this GTT (E). **(G)** Serum insulin measurements performed in 12-hour–fasted mice (3m) during a GTT (E). **(H)** ITT performed on 12-hour–fasted 3-month-old control and RL-m155 mice. **(I)** AUC calculated from mice in (H). **(J)** ITT of 12-hour–fasted control and RL-m155 mice. **(K)** AUC analysis for this ITT (J). Values are statistically significant at *P<0.05; **P<0.01 and ^#^*P*<0.001.
(TIF)

S4 Fig Glucose metabolism in RL-155 mice fed an HFD. **(A)** Blood glucose concentrations in 24-hour-fasted control (Con) and RL-155 mice (7m) maintained on chow or an HFD (high-fat diet) (60% fat). **(B)** GTT in 12-hour–fasted mice (maintained on chow or an HFD). **(C)** ITT performed on 12-hour–fasted 7-month-old control and RL-m155 mice maintained on chow or an HFD. 28-week-old mice were maintained on chow or were fed an HFD beginning at age 12 wk. For RL-155 mice vs. control mice fed chow: *P <0.05, **P<0.01, ***P<0.001; for RL-155 mice vs. control mice fed a high-fat diet: ^#^*P*<0.05, ^##^*P*<0.01, ^###^*P*<0.001.
(TIF)

S5 Fig Western blot analysis of PDK4 expression in liver, WAT and skeletal muscle (SM) of RL-m155 mice. (TIF)

S6 Fig Insulin-stimulated Akt phosphorylation in hepa1-6 cells in the absence (miR-con) or presence of miR-155 mimics. Hepa1-6 cells transfected with miR-155 mimics or mimics control were challenged with human insulin at 50 IU/L or 100 IU/L for 0, 15 and 30min, respectively. Protein levels of total and phosphorylated AKT were detected by Western blotting. The results were obtained from three independent experiments and a representative immunoblot is shown.
(TIF)

S7 Fig SOCS1 is a direct target of miR-155. (**A**) Sequence alignment of 3’-UTR of human (Hsa), mouse (Mmu), rhesus (Mml) and rat (Rno) SOCS1 highlighting miR-155 binding site. (**B**) Sequence alignment of 3’-UTR of human and mouse SOCS1 with respect to miR-155 mature sequence, showing possible interaction through a conserved binding site. (**C**) Immunoblot analysis of SOCS1 expression in liver of RL-m155 transgenic mice and KO mice.
(TIF)

S8 Fig qRT-PCR analysis of C/EBPβ, HDAC4 and PDK4 expression in hepa1-6 cells transfected with the indicated siRNA oligonucleotides. SCR: scrambled siRNA. *, *P* < 0.05 compared with siSCR.
(TIF)

S9 Fig (Extended Data Fig 6F) The fold changes in ^18^F-FDG uptake between miR-155-expressing indicated cells and the corresponding control cells. Data are presented as fold changes in the miR-155-expressing cells compared to the control cells. UC: untransfected cells. *P < 0.05; **P < 0.01; NS, not significant.
(TIF)

S10 Fig (Extended Data Fig 7G) The fold changes in ^18^F-FDG uptake between siRNA-transfected hepa1-6 cells and control cells. Data are presented as fold changes in siRNA-transfected cells compared to control cells. UC: untransfected cells. *P < 0.05; **P < 0.01; NS, not significant.
(TIF)

S11 Fig Proposed model for the role of miR-155 in known molecular pathways crucial for improved glucose metabolism. (TIF)

S1 Table Primers for qRT-PCR analysis of miRNAs. (DOC)

S2 Table Primers for qRT-PCR analysis of insulin sensitivity-related human genes expression. (DOC)

S3 Table Primers for qRT-PCR analysis of glucose metabolism and insulin sensitivity-related mouse genes expression. (DOC)

S4 Table List of antibodies and suppliers used for immunoblotting and immunohistochemistry. (DOC)

S5 Table The verified or putative miR-155 target genes implicated in insulin signaling, glucose metabolism and diabetes. (DOC)

S1 Data Experiment-level data of Fig 1. (XLS)

S2 Data Experiment-level data of Fig 2. (XLS)

S3 Data Experiment-level data of Fig 3. (XLS)

S4 Data Experiment-level data of Fig 4. (XLS)

S5 Data Experiment-level data of Fig 5. (XLS)

S6 Data Experiment-level data of Fig 6. (XLS)
