# Supplementary figures and images for: MiR-155 Enhances Insulin Sensitivity by Coordinated Regulation of Multiple Genes in Mice
Source: PLoS Genet. 2016 Oct 6;12(10):e1006308. doi: 10.1371/journal.pgen.1006308 (PMC5053416; doi:10.1371/journal.pgen.1006308)

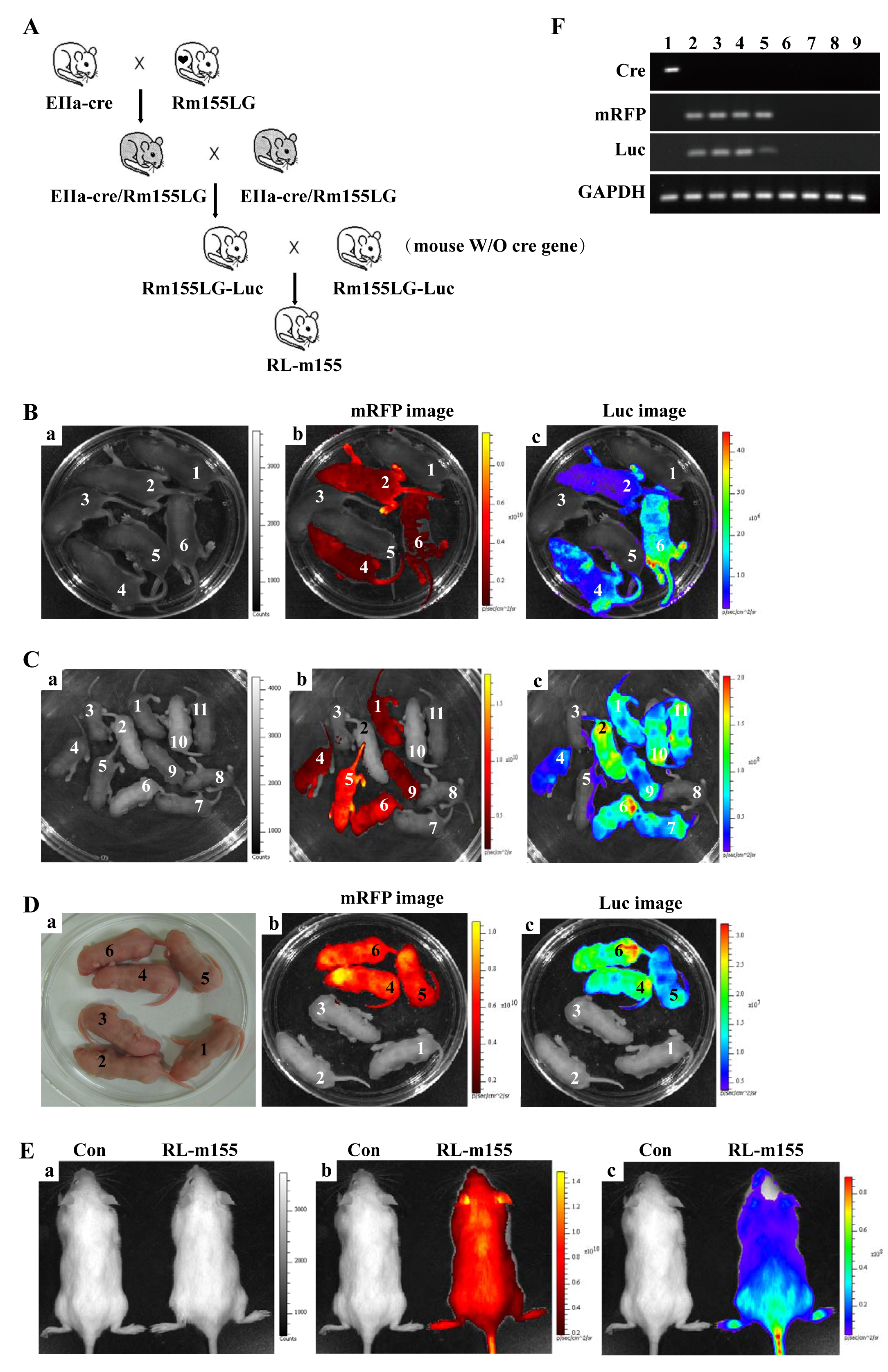

Supplement: S1 Fig — (A) Schematic strategy for generating RL-m155 transgenic mice. Procedure for producing transgenic mice which can globally overexpress mouse miR-155 transgene in multiple organs and tissues of mice was detailedly illustrated in S1A Fig. Briefly, at 6–8 wk of age, the heterozygous Rm155LG transgenic mice[8] were mated with the homozygous EIIa-Cre transgenic mice (FVB/N-Tg(EIIa-cre)C5379Lmgd/J)[65] to generate F1 (S1B Fig); next, both luciferase (Luc)- and mRFP-positive F1 animals (2#, 4# or 6#) (shown in S1B-b,c Fig) were intercrossed to produce F2 (shown in S1C Fig), including Luc- and mRFP-positive F2 animals with white fur (6#). Finally, both Luc- and mRFP-positive F2 animals with white fur and without Cre gene (determined by PCR-based genotyping) were intercrossed to produce RL-m155 transgenic mice (R: mRFP; L: Luc) (S1D Fig) which can globally and constantly express mRFP (S2A Fig), Luc (S2A Fig) and miR-155 (S2B Fig) transgenes in multiple organs and tissues of RL-m155 transgenic mice. Additionally, the genetic background of RL-m155 mice is FVB/N strain. (B) Whole-body fluorescence (b) and bioluminescence (c) imaging for newborn offspring derived from mating heterozygous Rm155LG transgenic mice with homozygous EIIa-Cre mice. (C) In vivo mRFP (b) and luc (c) imaging for newborn offspring derived from intercrossing of both Luc- and mRFP-positive F1 animals (i.e., 2#, 4# or 6#) (shown in S1B-b,c Fig). (D) Whole-body fluorescence (b) and bioluminescence (c) imaging for newborn RL-m155 transgenic mice. Both Luc- and mRFP-positive mice (i.e., 4#, 5# and 6#) (shown in S1D Fig) are RL-m155 transgenic mice. (E) Whole-body fluorescence (b) and bioluminescence (c) imaging for adult RL-m155 transgenic mice. (F) PCR-based genotyping for Cre, mRFP and Luc transgenes in RL-m155 transgenic mice. These RL-m155 transgenic mice (shown in S1D Fig) were individually analyzed by PCR for the genomic integration of Cre, mRFP and Luc transgenes with tail biopsy-derived DNA. PCR products [file pgen.1006308.s001.tif]

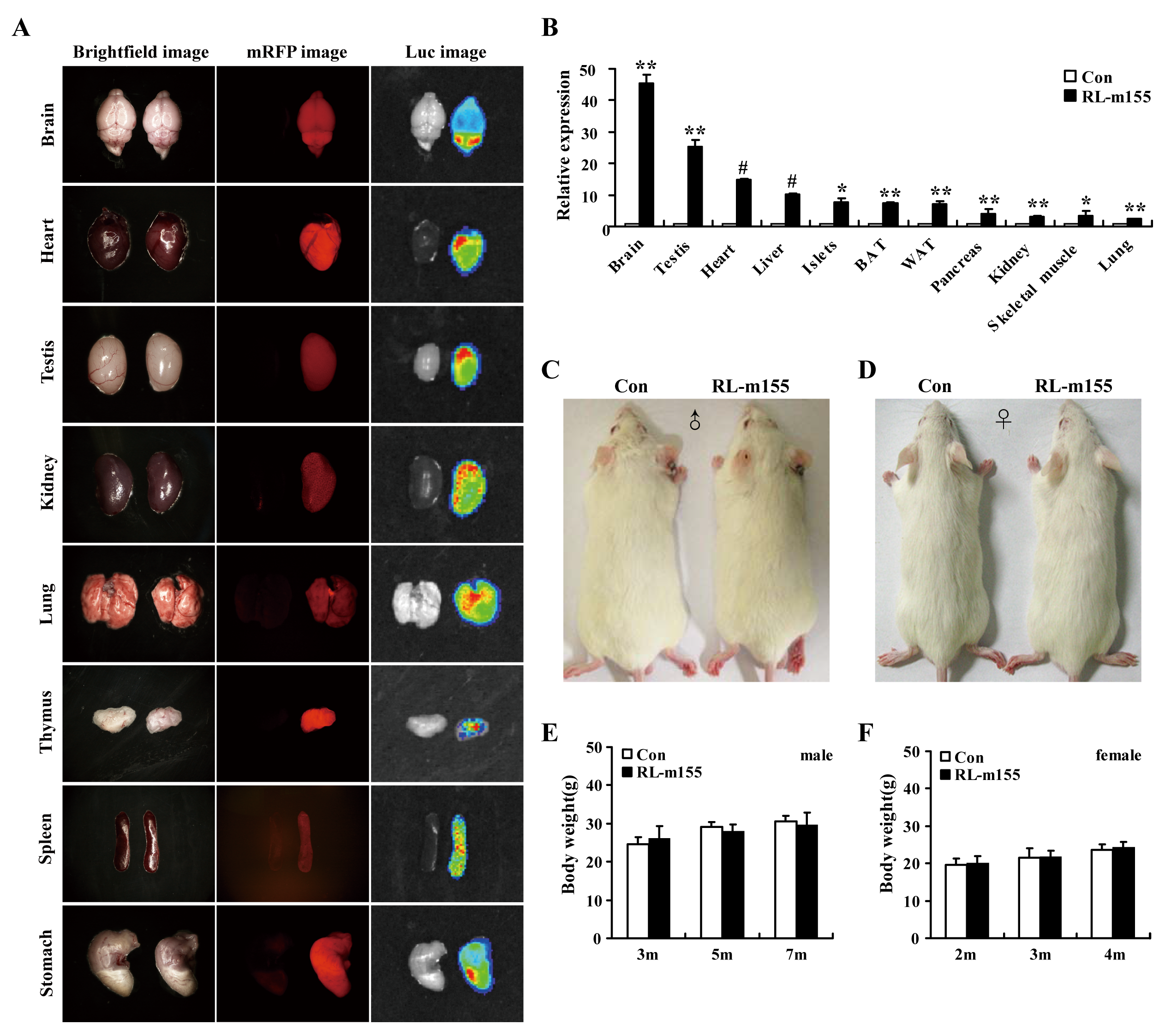

Supplement: S2 Fig — (A) mRFP and Luc expression in multiple organs and tissues of RL-m155 transgenic mice. The left organ samples in each figure were obtained from one control littermate, while the right organ samples in each figure were isolated from one RL-m155 transgenic mouse. mRFP expression in the postnatal organs and tissues of RL-m155 transgenic mice was assayed under stereo fluorescent microscope (Nikon, AZ100), while bioluminescence imaging for multiple organs and tissues obtained from RL-m155 transgenic mouse and littermate controls was measured noninvasively using the IVIS LuminaIIimaging system (Xenogen Corp., Alameda, CA). Muscle and pancreas from RL-m155R transgenic mice (the right samples in each figure) can be distinguished from their wildtype littermates according to their deep red color under daylight (Fig 2A). (B) qRT-PCR analysis of the expression of miR-155 transgene in multiple organs and tissues of RL-m155 transgenic mice. BAT: brown adipose tissue; WAT: white adipose tissue. (C) RL-m155 (right) and control (left) male littermates at 20 weeks of age. (D) RL-m155 (right) and control (left) female littermates at age 12 weeks. (E-F) Body weight of control mice vs. RL-m155 transgenic mice at different ages. Values are mean ± SD; n = 5–10 mice per time point. *, P < 0.05 compared with control mice;**, P<0.01 compared with control mice;#, P<0.001 compared with control mice. (TIF) [file pgen.1006308.s002.tif]

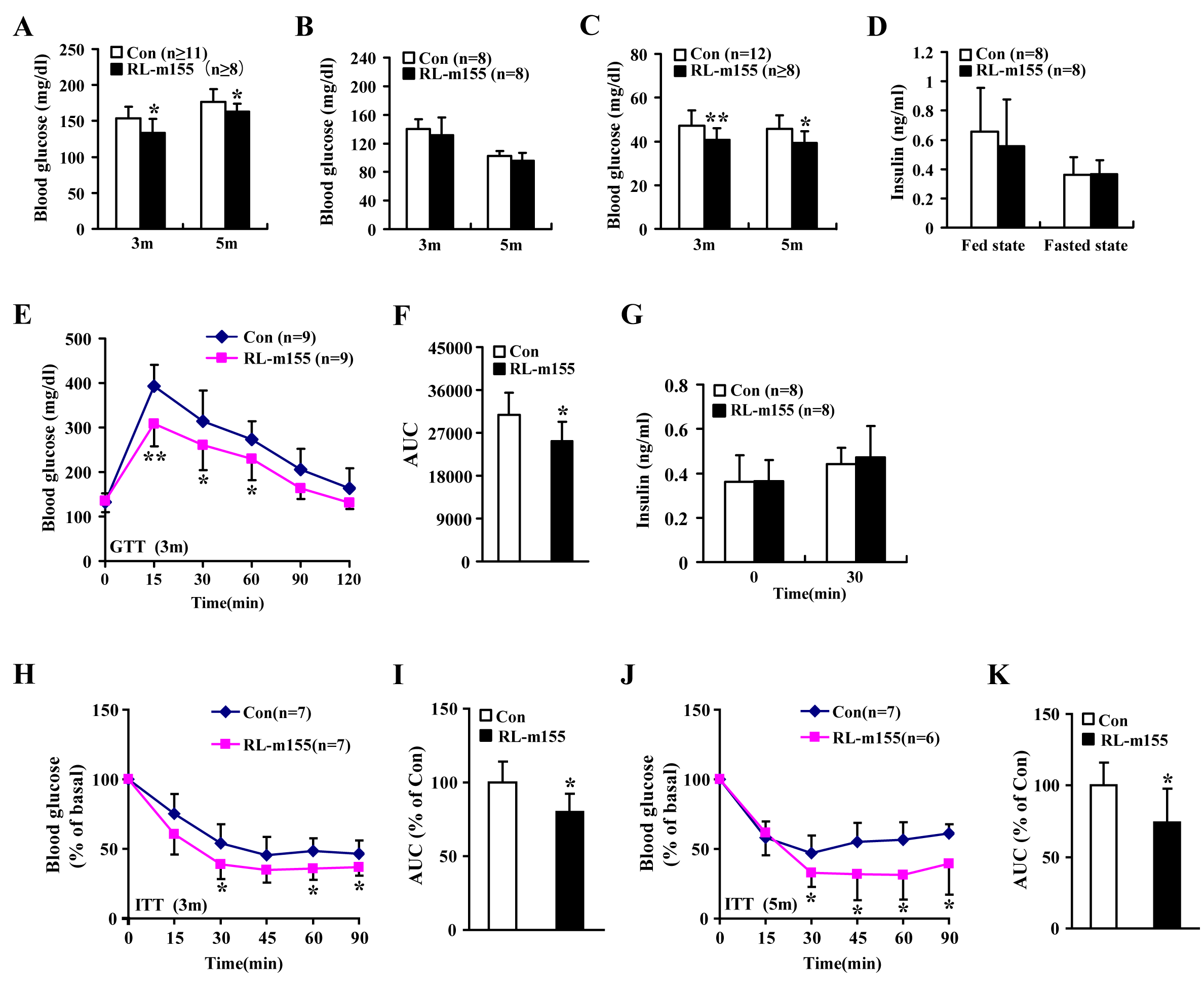

Supplement: S3 Fig — (A-C) Blood glucose concentrations in fed-state (A), 12-hour–fasted (B) and 24-hour–fasted (C) mice at indicated ages. (D) Serum insulin concentrations in fed-state and 12-hour–fasted mice (5m). (E-F) GTT in 12-hour–fasted mice (E) and AUC (F) for this GTT (E). (G) Serum insulin measurements performed in 12-hour–fasted mice (3m) during a GTT (E). (H) ITT performed on 12-hour–fasted 3-month-old control and RL-m155 mice. (I) AUC calculated from mice in (H). (J) ITT of 12-hour–fasted control and RL-m155 mice. (K) AUC analysis for this ITT (J). Values are statistically significant at *P<0.05; **P<0.01 and #P<0.001. (TIF) [file pgen.1006308.s003.tif]

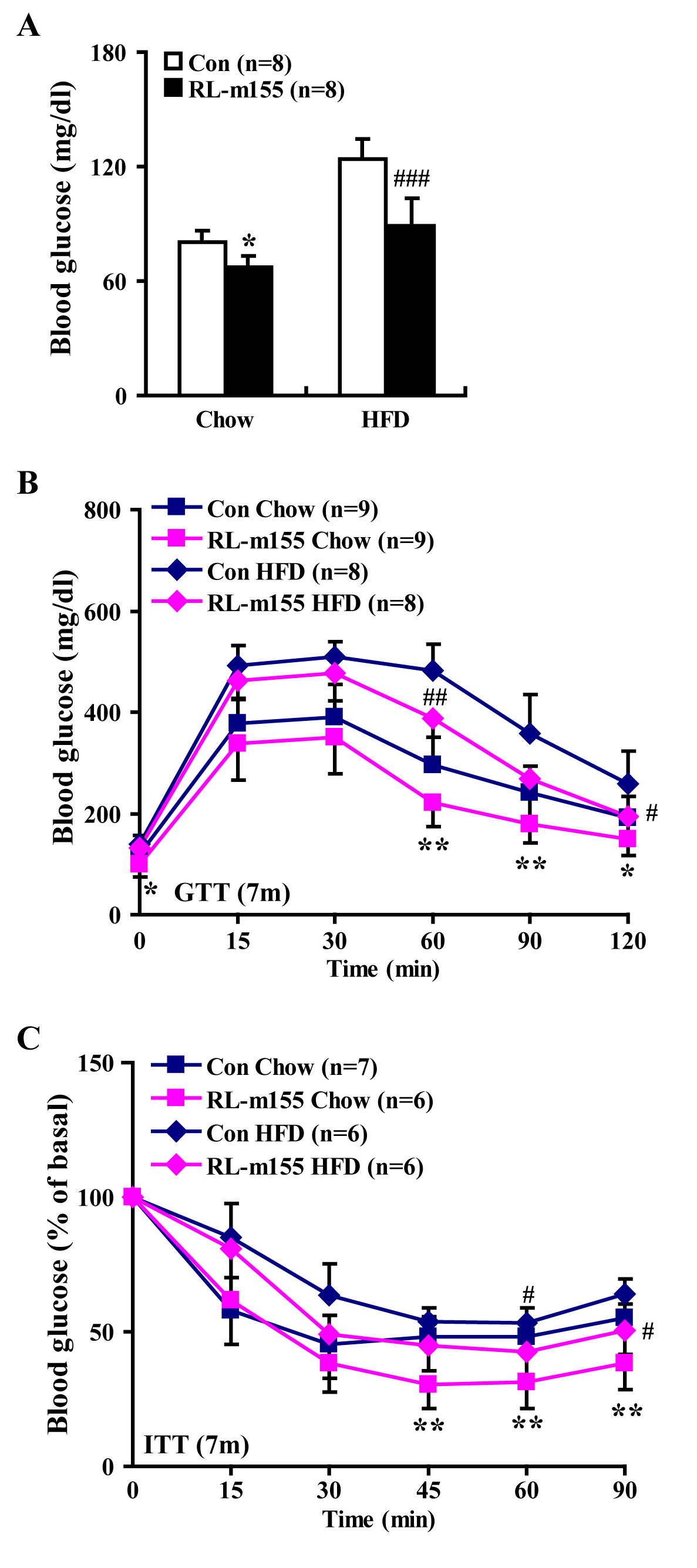

Supplement: S4 Fig — (A) Blood glucose concentrations in 24-hour-fasted control (Con) and RL-155 mice (7m) maintained on chow or an HFD (high-fat diet) (60% fat). (B) GTT in 12-hour–fasted mice (maintained on chow or an HFD). (C) ITT performed on 12-hour–fasted 7-month-old control and RL-m155 mice maintained on chow or an HFD. 28-week-old mice were maintained on chow or were fed an HFD beginning at age 12 wk. For RL-155 mice vs. control mice fed chow: *P <0.05, **P<0.01, ***P<0.001; for RL-155 mice vs. control mice fed a high-fat diet: #P<0.05, ##P<0.01, ###P<0.001. (TIF) [file pgen.1006308.s004.tif]

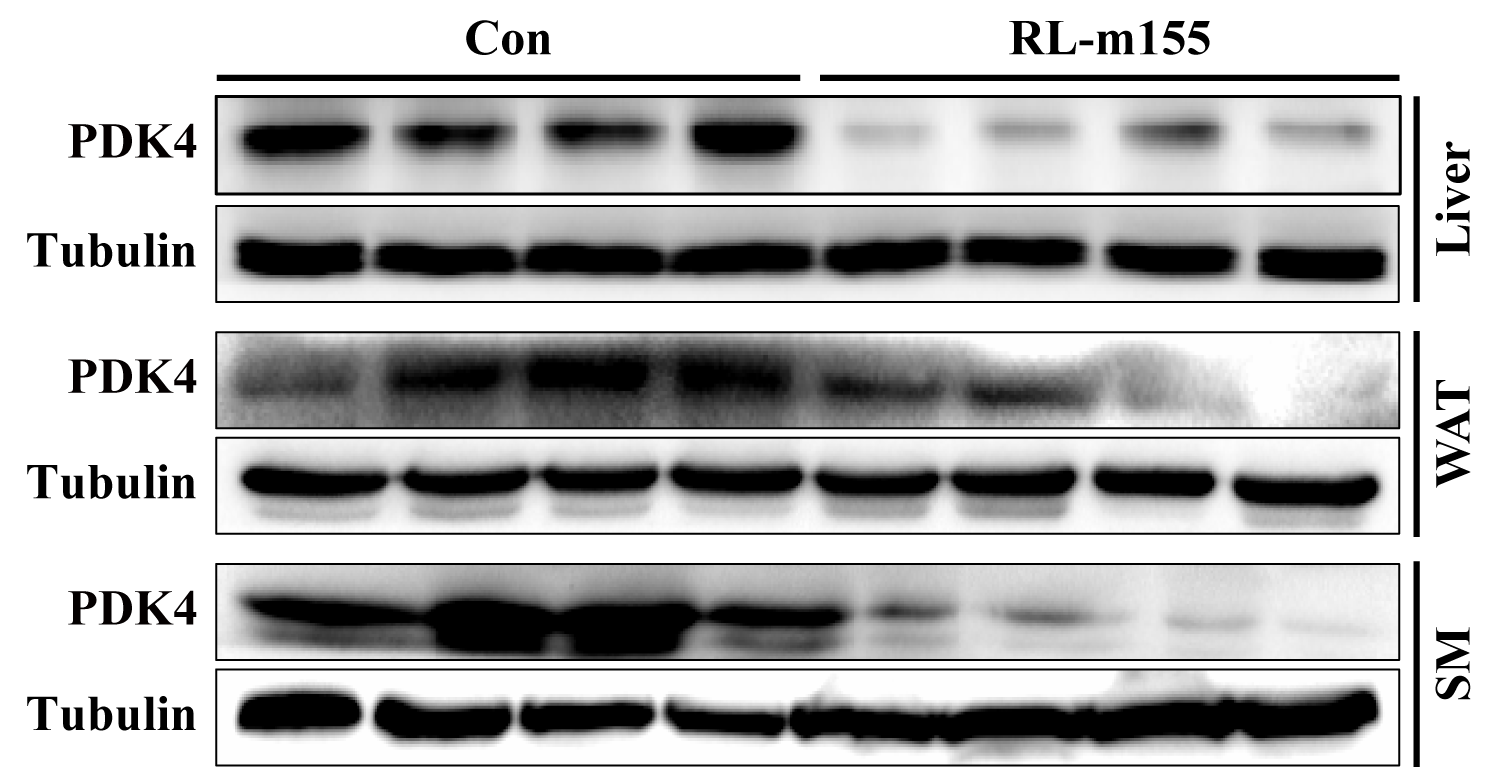

Supplement: S5 Fig — (TIF) [file pgen.1006308.s005.tif]

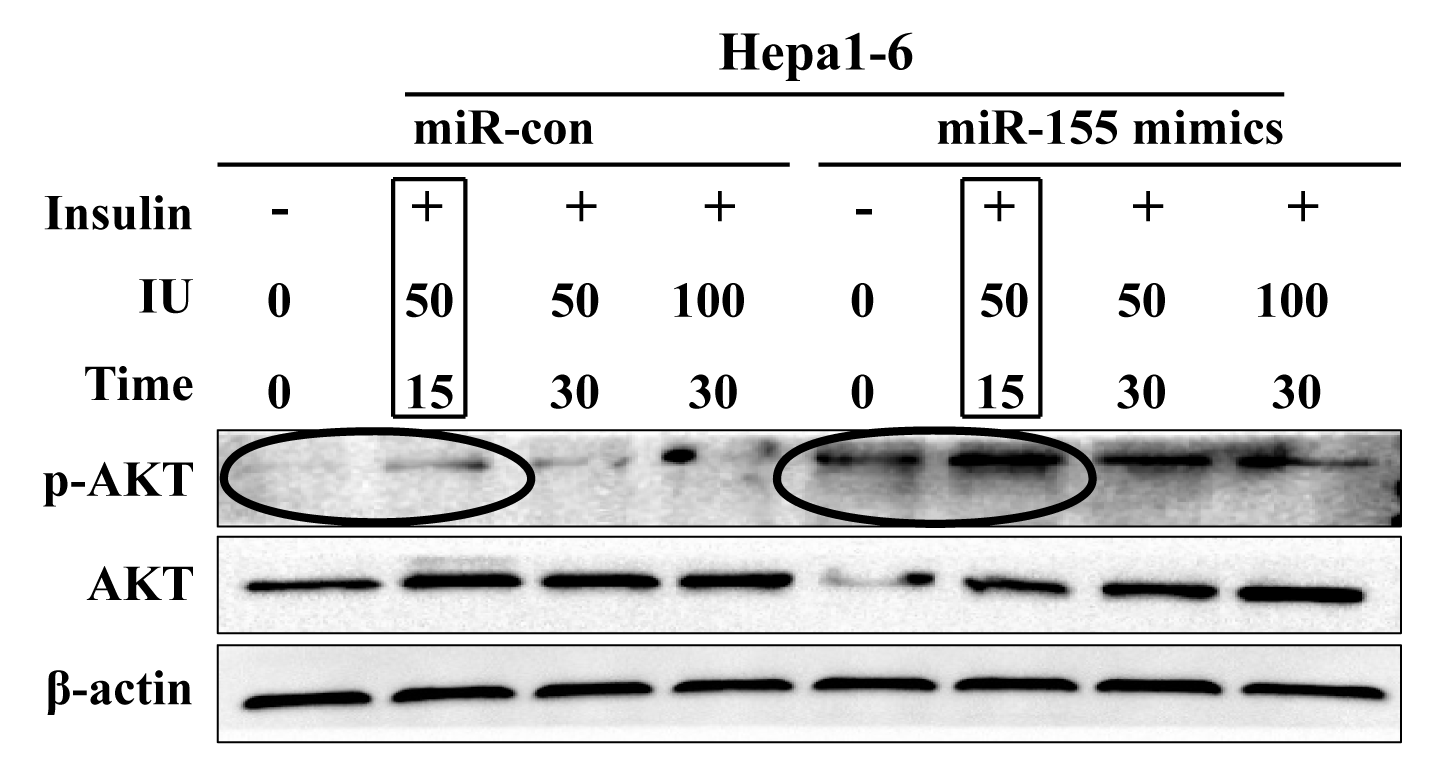

Supplement: S6 Fig — Hepa1-6 cells transfected with miR-155 mimics or mimics control were challenged with human insulin at 50 IU/L or 100 IU/L for 0, 15 and 30min, respectively. Protein levels of total and phosphorylated AKT were detected by Western blotting. The results were obtained from three independent experiments and a representative immunoblot is shown. (TIF) [file pgen.1006308.s006.tif]

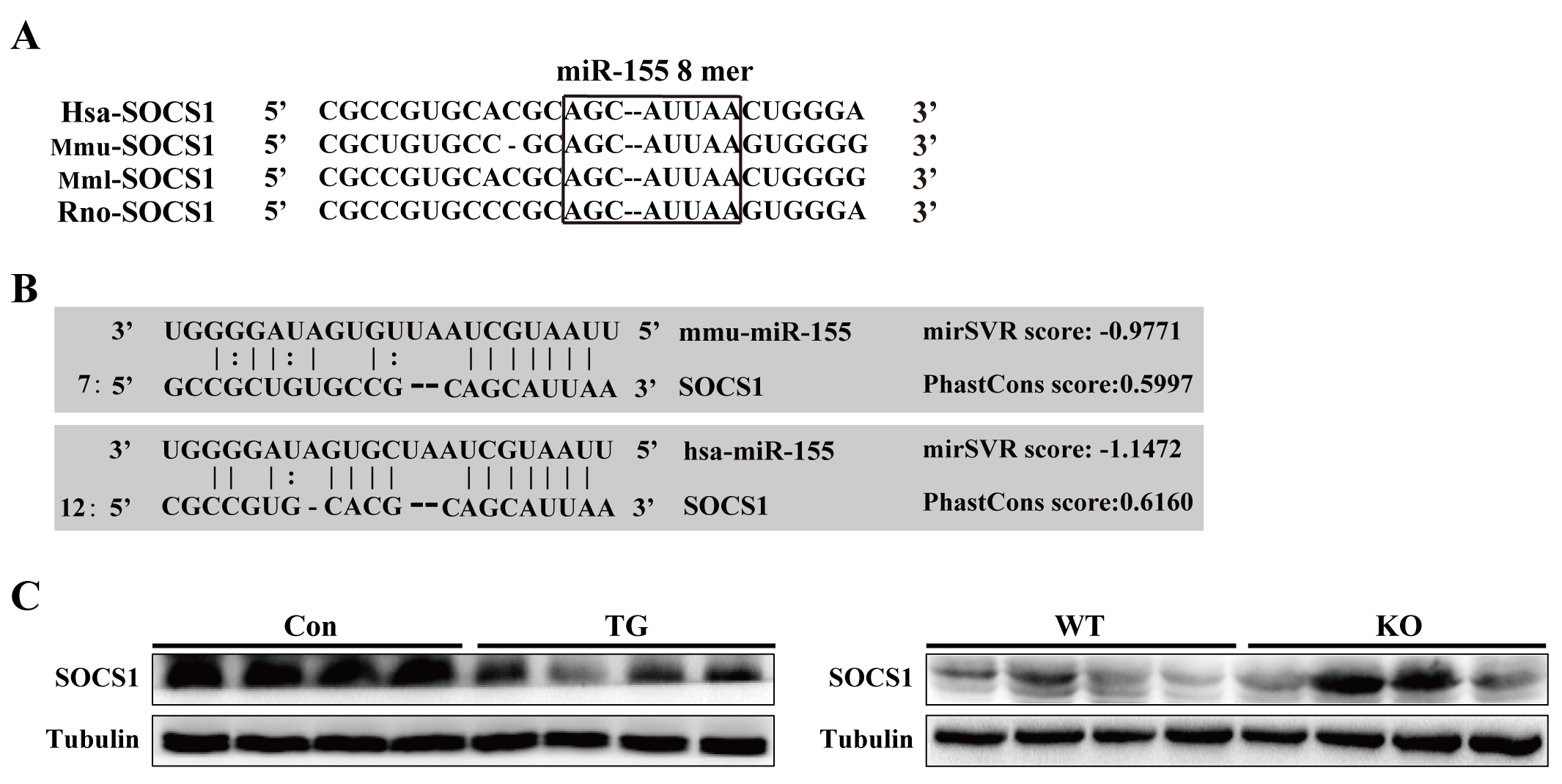

Supplement: S7 Fig — (A) Sequence alignment of 3’-UTR of human (Hsa), mouse (Mmu), rhesus (Mml) and rat (Rno) SOCS1 highlighting miR-155 binding site. (B) Sequence alignment of 3’-UTR of human and mouse SOCS1 with respect to miR-155 mature sequence, showing possible interaction through a conserved binding site. (C) Immunoblot analysis of SOCS1 expression in liver of RL-m155 transgenic mice and KO mice. (TIF) [file pgen.1006308.s007.tif]

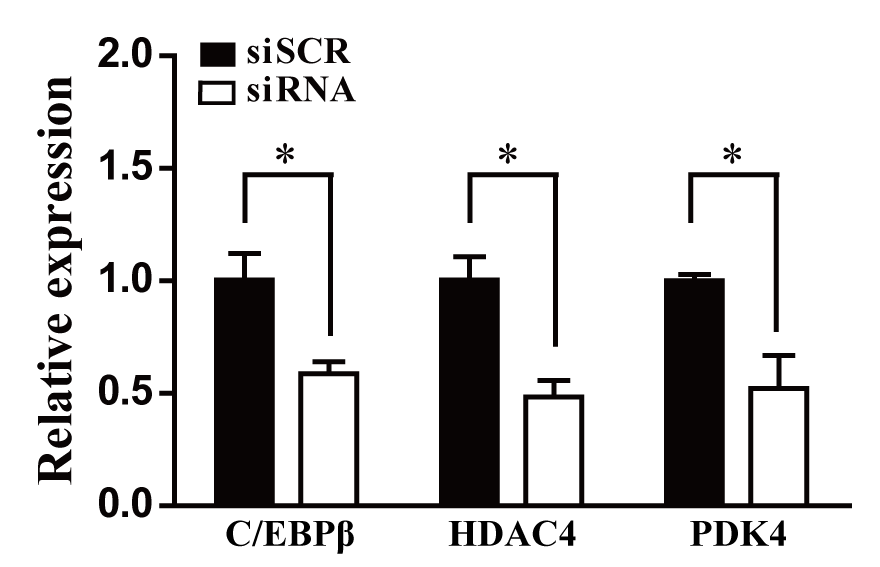

Supplement: S8 Fig — SCR: scrambled siRNA. *, P < 0.05 compared with siSCR. (TIF) [file pgen.1006308.s008.tif]

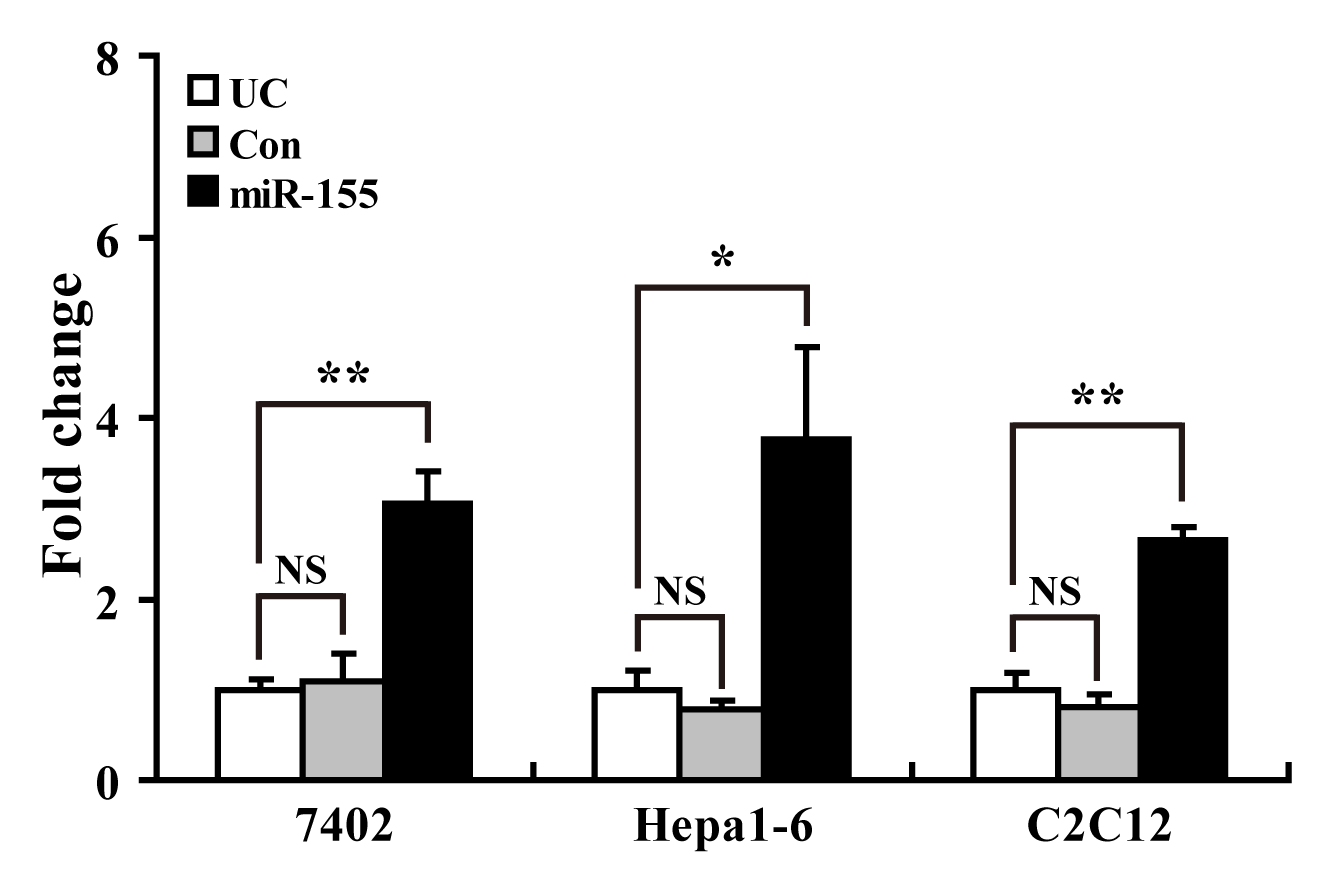

Supplement: S9 Fig — Data are presented as fold changes in the miR-155-expressing cells compared to the control cells. UC: untransfected cells. *P < 0.05; **P < 0.01; NS, not significant. (TIF) [file pgen.1006308.s009.tif]

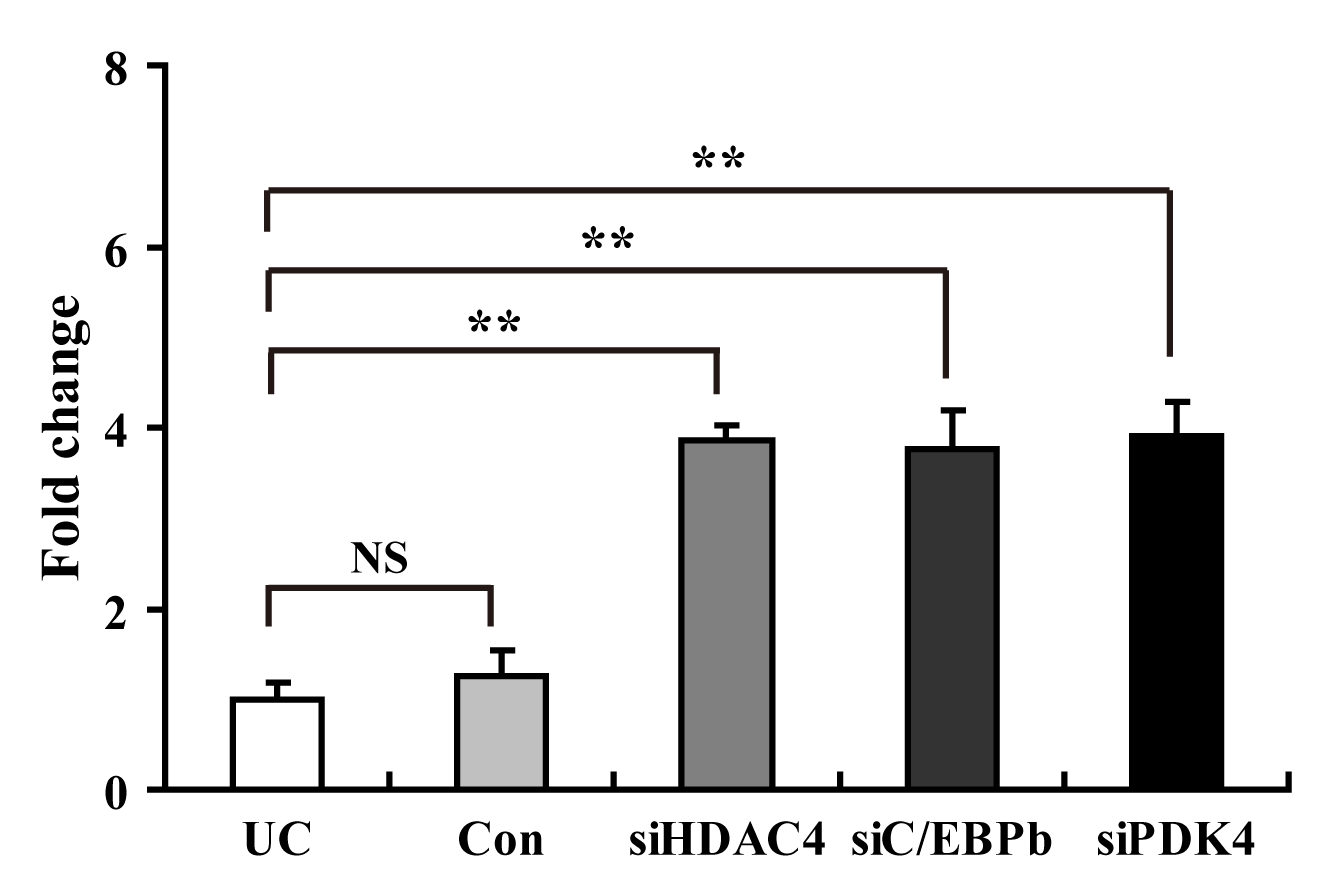

Supplement: S10 Fig — Data are presented as fold changes in siRNA-transfected cells compared to control cells. UC: untransfected cells. *P < 0.05; **P < 0.01; NS, not significant. (TIF) [file pgen.1006308.s010.tif]

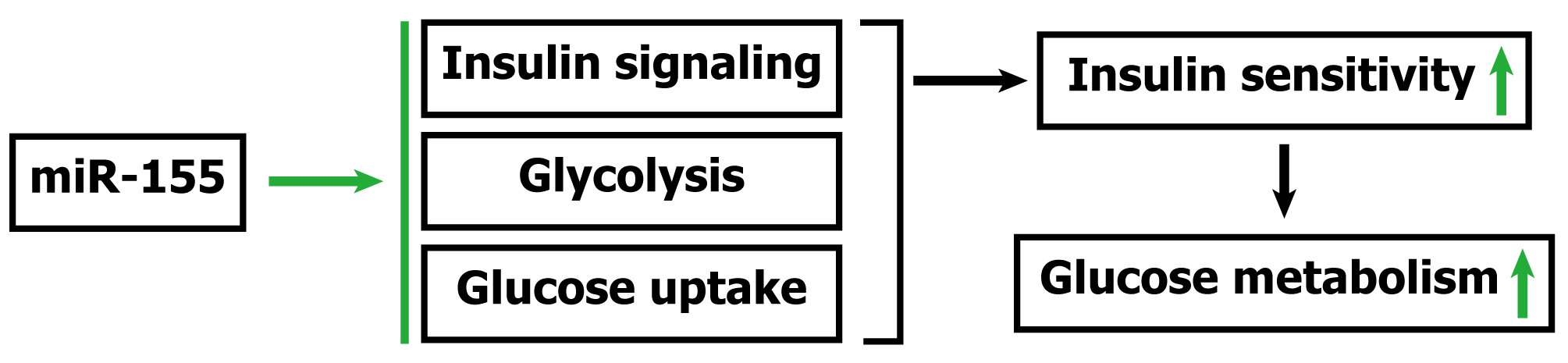

Supplement: S11 Fig — (TIF) [file pgen.1006308.s011.tif]
