# Supplementary material for: MiR-155 Enhances Insulin Sensitivity by Coordinated Regulation of Multiple Genes in Mice
Source: PLoS Genet. 2016 Oct 6;12(10):e1006308. doi: 10.1371/journal.pgen.1006308 (PMC5053416; doi:10.1371/journal.pgen.1006308)
Supplement: S1 Table — (DOC) [file pgen.1006308.s012.doc]

**S1 Table Primers for qRT-PCR analysis of miRNAs**

| **Primer name** | **Primer sequence** |
| --- | --- |
| U6 snRNA-RT | AACGCTTCACGAATTTGCGT |
| U6 snRNA-forward primer | CTCGCTTCGGCAGCACA |
| U6 snRNA-reverse primer | AACGCTTCACGAATTTGCGT |
| miR-155 RT primer | GTCGTATCCAGTGCAGGGTCCGAGGTATTCGCACTGGATACGACACCCC |
| mmu-miR155-forward primer | ACTGTTAATGCTAATtGTGATAGG |
| mmu-miR155-reverse primer | GTGCAGGGTCCGAGGTATTC |
| hsa-miR-155-forward primer | GACTGTTAATGCTAATCGTGATAG |
| hsa-miR-155-reverse primer | GTGCAGGGTCCGAGGTATTC |
| hsa-miR-107 RT primer | GTCGTATCCAGTGCAGGGTCCGAGGTATTCGCACTGGATACGACTGATA |
| hsa-miR-107-forward primer | AGCAGCATTGTACAGGGCTATCA / AGCAGCATTGTACAGGGCTA |
| hsa-miR-107-reverse primer | GTGCAGGGTCCGAGGTATTC |
| hsa-miR-146a RT primer | GTCGTATCCAGTGCAGGGTCCGAGGTATTCGCACTGGATACGACAACCC |
| hsa-miR-146a-forward primer | TGAGAACTGAATTCCATGGGTT |
| hsa-miR-146a-reverse primer | GTGCAGGGTCCGAGGTATTC |
| hsa-miR-451a RT primer | GTCGTATCCAGTGCAGGGTCCGAGGTATTCGCACTGGATACGACAACTC |
| hsa-miR-451a-forward primer | CTGAAACCGTTACCATTACTG |
| hsa-miR-451a-reverse primer | GTGCAGGGTCCGAGGTATTC |
