# Supplementary material for: MiR-155 Enhances Insulin Sensitivity by Coordinated Regulation of Multiple Genes in Mice
Source: PLoS Genet. 2016 Oct 6;12(10):e1006308. doi: 10.1371/journal.pgen.1006308 (PMC5053416; doi:10.1371/journal.pgen.1006308)
Supplement: S2 Table — (DOC) [file pgen.1006308.s013.doc]

**S2 Table Primers for qRT-PCR analysis of**

**insulin sensitivity-related human genes expression**

| **Gene** | **Forward Primer (5’-3’)** | **Reverse Primer (5’-3’)** |
| --- | --- | --- |
| β-actin | CCCAAGGCCAACCGCGAGAAGAT | GTCCCGGCCAGCCAGGTCCAG |
| C/EBPβ | CTTCAGCCCGTACCTGGAG | GGAGAGGAAGTCGTGGTGC |
| HDAC4 | AGCGTCCGTTGGATGTCAC | CCTTCTCGTGCCACAAGTCT |
| PDK4 | AACACCAGGAAAATCAGCC | AAAACCAGCCAAAGGAGC |
| PTEN | TTTGAAGACCATAACCCACCAC | ATTACACCAGTTCGTCCCTTTC |
| SOCS1 | TTTTCGCCCTTAGCGTGAAGA | GAGGCAGTCGAAGCTCTCG |
| SOCS3 | CCTGCGCCTCAAGACCTTC | GTCACTGCGCTCCAGTAGAA |
