# Supplementary material for: MiR-155 Enhances Insulin Sensitivity by Coordinated Regulation of Multiple Genes in Mice
Source: PLoS Genet. 2016 Oct 6;12(10):e1006308. doi: 10.1371/journal.pgen.1006308 (PMC5053416; doi:10.1371/journal.pgen.1006308)
Supplement: S3 Table — (DOC) [file pgen.1006308.s014.doc]

**S3 Table Primers for qRT-PCR analysis of glucose metabolism**

**and insulin sensitivity-related mouse genes expression**

| **Gene** | **Forward Primer (5’-3’)** | **Reverse Primer (5’-3’)** |
| --- | --- | --- |
| β-actin | CTGGCCGGGACCTGACAGACTACC | ATCGGAACCGCTCGTTGCCAAT |
| ATF4 | ATGGCGCTCTTCACGAAATC | ACTGGTCGAAGGGGTCATCAA |
| C/EBPβ | GGAGACGCAGCACAAGGT | AGCTGCTTGAACAAGTTCCG |
| Fabp5 | TGAAAGAGCTAGGAGTAGGACTG | CTCTCGGTTTTGACCGTGATG |
| Gck | TGAGCCGGATGCAGAAGGA | GCAACATCTTTACACTGGCCT |
| GLUT1 | CAGTTCGGCTATAACACTGGTG | GCCCCCGACAGAGAAGATG |
| GLUT2 | TCAGAAGACAAGATCACCGGA | GCTGGTGTGACTGTAAGTGGG |
| GLUT4 | GTGACTGGAACACTGGTCCTA | CCAGCCACGTTGCATTGTAG |
| HDAC4 | CTGCAAGTGGCCCCTACAG | CTGCTCATGTTGACGCTGGA |
| PDK4 | TTTGGTGGAGTTCCATGAGAA | GAACTTTGACCAGCGTGTCT |
| PKM2 | GCCGCCTGGACATTGACTC | CCATGAGAGAAATTCAGCCGAG |
| PTEN | TGGATTCGACTTAGACTTGACCT | GCGGTGTCATAATGTCTCTCAG |
| SOCS1 | CTGCGGCTTCTATTGGGGAC | AAAAGGCAGTCGAAGGTCTCG |
| SOCS3 | ATGGTCACCCACAGCAAGTTT | TCCAGTAGAATCCGCTCTCCT |
