# Supplementary material for: MiR-155 Enhances Insulin Sensitivity by Coordinated Regulation of Multiple Genes in Mice
Source: PLoS Genet. 2016 Oct 6;12(10):e1006308. doi: 10.1371/journal.pgen.1006308 (PMC5053416; doi:10.1371/journal.pgen.1006308)
Supplement: S4 Table — (DOC) [file pgen.1006308.s015.doc]

**S4 Table List of antibodies and suppliers used**

**for immunoblotting and immunohistochemistry**

| **Antibody** | **Isotype** | **Suppliers** |
| --- | --- | --- |
| Ki67 | Rabbit | Abcam |
| SOCS1 | Rabbit | Abcam |
| Tubulin | Rabbit | Bioworld Technology |
| β-actin | Rabbit | Cell Signaling |
| PTEN | Rabbit | Cell Signaling |
| Insulin | Rabbit | Cell Signaling |
| Glucagon | Rabbit | Cell Signaling |
| AKT | Rabbit | Cell Signaling |
| p-AKT (Ser473) | Rabbit | Cell Signaling |
| IRS-1 | Rabbit | Cell Signaling |
| p- IRS-1(Ser307) | Rabbit | Cell Signaling |
| BrdU | Mouse | GE Healthcare |
| SOCS3 | Rabbit | Proteintech |
| PDK4 | Rabbit | Proteintech |
| HDAC4 | Rabbit | SANTA CRUZ |
| C/EBPβ | Rabbit | SANTA CRUZ |
