# Supplementary material for: MiR-155 Enhances Insulin Sensitivity by Coordinated Regulation of Multiple Genes in Mice
Source: PLoS Genet. 2016 Oct 6;12(10):e1006308. doi: 10.1371/journal.pgen.1006308 (PMC5053416; doi:10.1371/journal.pgen.1006308)
Supplement: S5 Table — (DOC) [file pgen.1006308.s016.doc]

**S5 Table The verified or putative miR-155 target genes**

**implicated in insulin signaling, glucose metabolism and diabetes**

| **Human** | | **Mouse** | **Functions** | **References** |
| --- | --- | --- | --- | --- |
| AGTR1 | AGTR1 | | **√** | [PMID: 25339158](http://www.ncbi.nlm.nih.gov/pubmed/25339158), [24599011](http://www.ncbi.nlm.nih.gov/pubmed/24599011), [24587998](http://www.ncbi.nlm.nih.gov/pubmed/24587998), [24371439](http://www.ncbi.nlm.nih.gov/pubmed/24371439) |
| APC | APC | | **√** | [PMID: 24559924](http://www.ncbi.nlm.nih.gov/pubmed/24559924), [24560421](http://www.ncbi.nlm.nih.gov/pubmed/24560421), 24660539, 22447930 |
| ARID2 | ARID2 | | **—** |  |
| BACH1 | BACH1 | | **√** | [PMID: 23880309](http://www.ncbi.nlm.nih.gov/pubmed/23880309) |
| CEBPB | CEBPB | | **√** | [PMID: 17387171](http://www.ncbi.nlm.nih.gov/pubmed/17387171), 10747954, 17620318 |
| CES3 | CES3 | | **√** | [PMID: 24362705](http://www.ncbi.nlm.nih.gov/pubmed/24362705), 22707181 |
| CYR61 | CYR61 | | **√** | [PMID: 24553917](http://www.ncbi.nlm.nih.gov/pubmed/24553917), [17333105](http://www.ncbi.nlm.nih.gov/pubmed/17333105) |
| DET1 | DET1 | | **—** |  |
| EDN1 | EDN1 | | **√** | [PMID: 25091502](http://www.ncbi.nlm.nih.gov/pubmed/25091502), [24989952](http://www.ncbi.nlm.nih.gov/pubmed/24989952), [24955982](http://www.ncbi.nlm.nih.gov/pubmed/24955982), [24769306](http://www.ncbi.nlm.nih.gov/pubmed/24769306), [24394957](http://www.ncbi.nlm.nih.gov/pubmed/24394957) |
| ETS1 | ETS1 | | **√** | [PMID: 23756832](http://www.ncbi.nlm.nih.gov/pubmed/23756832), 20598359, 19806240, [17708355](http://www.ncbi.nlm.nih.gov/pubmed/17708355) |
| FADD | FADD | | **√** | [PMID: 25078620](http://www.ncbi.nlm.nih.gov/pubmed/25078620), 10982176 |
| FGF7 | FGF7 | | **√** | [PMID: 17951544](http://www.ncbi.nlm.nih.gov/pubmed/17951544) |
| FOXO3 | FOXO3 | | **√** | [PMID: 25166345](http://www.ncbi.nlm.nih.gov/pubmed/25166345), 24013118, 22187289 |
| HDAC4 | HDAC4 | | **√** | [PMID: 19793100](http://www.ncbi.nlm.nih.gov/pubmed/19793100), 24086512, 24768298 |
| HIVEP2 | HIVEP2 | | **—** |  |
| IFNGR1 | IFNGR1 | | **√** | [PMID: 22344559](http://www.ncbi.nlm.nih.gov/pubmed/22344559) |
| IKBKE | IKBKE | | **√** | [PMID: 19737522](http://www.ncbi.nlm.nih.gov/pubmed/19737522) |
| INPP5D | INPP5D | | **—** |  |
| IRAK3 | IRAK3 | | **√** | [PMID: 24696448](http://www.ncbi.nlm.nih.gov/pubmed/24696448) |
| JARID2 | JARID2 | | **—** |  |
| LDOC1 | LDOC1 | | **—** |  |
| MAFB | MAFB | | **√** | [PMID: 20581830](http://www.ncbi.nlm.nih.gov/pubmed/20581830) |
| MATR3 | MATR3 | | **—** |  |
| MECP2 | MECP2 | | **√** | [PMID: 25299635](http://www.ncbi.nlm.nih.gov/pubmed/25299635) |
| MEIS1 | MEIS1 | | **—** |  |
| MLH1 | MLH1 | | **—** |  |
| MSH2 | MSH2 | | **—** |  |
| MSH6 | MSH6 | | **—** |  |
| MYB | MYB | | **—** |  |
| MYO10 | MYO10 | | **—** |  |
| NFATC2IP | NFATC2IP | | **—** |  |
|  | PEA15A | | **—** |  |
| PELI1 | PELI1 | | **—** |  |
| PHF17 | PHF17 | | **—** |  |
| RHEB | RHEB | | **√** | [PMID: 21513702](http://www.ncbi.nlm.nih.gov/pubmed/21513702), 19258434, 12766776 |
| RHOA | RHOA | | **√** | [PMID: 25243854](http://www.ncbi.nlm.nih.gov/pubmed/25243854) |
| RIPK1 | RIPK1 | | **—** |  |
| RUNX2 | RUNX2 | | **√** | [PMID: 23806481](http://www.ncbi.nlm.nih.gov/pubmed/23806481), 21597275, 18162513 |
|  | SFPI1 | | **—** |  |
| SKI | SKI | | **—** |  |
| SMAD1 | SMAD1 | | **—** |  |
| SMAD2 | SMAD2 | | **√** | [PMID: 24595557](http://www.ncbi.nlm.nih.gov/pubmed/24595557), 24068386, 21976717 |
| SMAD5 | SMAD5 | | **√** | [PMID: 20079400](http://www.ncbi.nlm.nih.gov/pubmed/20079400) |
| SOCS1 | SOCS1 | | **√** | [PMID: 24966052](http://www.ncbi.nlm.nih.gov/pubmed/24966052), 24595859, 24002896, [23222907](http://www.ncbi.nlm.nih.gov/pubmed/23222907), 22861055 |
| SPI1 |  | | **√** | [PMID: 22454293](http://www.ncbi.nlm.nih.gov/pubmed/22454293) |
| TAB2 | TAB2 | | **—** |  |
| TM6SF1 | TM6SF1 | | **—** |  |
| TRF1 | TRF1 | | **√** | [PMID: 22170771](http://www.ncbi.nlm.nih.gov/pubmed/22170771) |
| TP53INP1 | TP53INP1 | | **√** | [PMID: 22046406](http://www.ncbi.nlm.nih.gov/pubmed/22046406) |
| TSHZ3 | TSHZ3 | | **—** |  |
| ZIC3 | ZIC3 | | **—** |  |
| ZNF652 | ZNF652 | | **—** |  |
|  | | | | |
| AAK1 | AAK1 | | **—** |  |
| AICDA | AICDA | | **—** |  |
| ASTN2 | ASTN2 | | **—** |  |
| BCORL1 | BCORL1 | | **—** |  |
| CARD11 | CARD11 | | **—** |  |
| CHD9 | CHD9 | | **—** |  |
| CSNK1G2 | CSNK1G2 | | **—** |  |
| DHX40 | DHX40 | | **—** |  |
| DYNC1I1 | DYNC1I1 | | **—** |  |
| G3BP2 | G3BP2 | | **—** |  |
| GDF6 | GDF6 | | **—** |  |
| H3F3A |  | | **—** |  |
| HNRNPA3 | HNRNPA3 | | **—** |  |
| ILF3 | ILF3 | | **√** | [PMID: 21347509](http://www.ncbi.nlm.nih.gov/pubmed/21347509) |
| KAT2A |  | | **—** |  |
| KRAS | KRAS | | **√** | [PMID: 24388967](http://www.ncbi.nlm.nih.gov/pubmed/24388967), 24178582, 22541435 |
| LPAR6 | LPAR6 | | **—** |  |
| MEF2A | MEF2A | | **√** | [PMID: 24303025](http://www.ncbi.nlm.nih.gov/pubmed/24303025), 22135324 |
| NDFIP1 | NDFIP1 | | **√** | [PMID: 24520172](http://www.ncbi.nlm.nih.gov/pubmed/24520172) |
| NOVA1 | NOVA1 | | **—** |  |
| PAM | PAM | | **—** |  |
| PLD5 | PLD5 | | **—** |  |
| RREB1 | RREB1 | | **√** | [PMID: 25027322](http://www.ncbi.nlm.nih.gov/pubmed/25027322) |
| SEC14L5 | SEC14L5 | | **—** |  |
| SEPT11 | SEPT11 | | **—** |  |
| SKIV2L2 | SKIV2L2 | | **—** |  |
| SOX1 | SOX1 | | **—** |  |
| STX16 | STX16 | | **—** |  |
| STXBP5L | STXBP5L | | **—** |  |
| TCEB1 | TCEB1 | | **—** |  |
| TLE4 | TLE4 | | **—** |  |
| TOMM20 | TOMM20 | | **—** |  |
| TTL | TTL | | **—** |  |
| USP14 | USP14 | | **—** |  |
|  | ZFP236 | | **—** |  |
|  | ZFP407 | | **—** |  |
| ZMYM2 |  | | **—** |  |
| ZNF236 |  | | **√** | [PMID: 12906866](http://www.ncbi.nlm.nih.gov/pubmed/12906866) |

“**√**” indicates these genes which are **verified to be** associated with insulin signalling, insulin sensitivity and resistance, glucose metabolism, and diabetes, while “**—**” shows these genes which are not yet reported to be involved in the aforementioned functions. **Red = experimentally verified target genes of miR-155; green = bioinformatically predicted target genes of miR-155.**
